# Unveiling the evolutionary relationships and the high cryptic diversity in Andean rainfrogs (Craugastoridae: *Pristimantis myersi* group)

**DOI:** 10.7717/peerj.14715

**Published:** 2023-03-01

**Authors:** Daniela Franco-Mena, Juan M. Guayasamin, Diego Andrade-Brito, Mario H. Yánez-Muñoz, Fernando J.M. Rojas-Runjaic

**Affiliations:** 1 Laboratorio de Biología Evolutiva, Instituto BIOSFERA, Colegio de Ciencias Biológicas y Ambientales COCIBA, Universidad San Francisco de Quito, Quito, Campus Cumbaya, Pichincha, Ecuador; 2 Facultad de Ciencias de Medio Ambiente, Universidad Tecnológica Indoamérica, Quito, Ecuador; 3 División de Herpetología, Instituto Nacional de Biodiversidad INABIO, Quito, Pichincha, Ecuador; 4 Fundación La Salle de Ciencias Naturales, Museo de Historia Natural La Salle (MHNLS), Caracas, Venezuela; 5 Laboratório de Herpetologia, Coordenação de Zoologia, Museu Paraense Emílio Goeldi (MPEG), Belém, Pará, Brazil

**Keywords:** Ecuador, *Pristimantis*, Andes, Evolutionary relationships, Cryptic diversity

## Abstract

**Background:**

*Pristimantis* is the most diverse genus of terrestrial frogs. Historically, it has been divided into several phenetic groups in order to facilitate species identification. However, in light of phylogenetic analysis, many of these groups have been shown to be non-monophyletic, denoting a high degree of morphological convergence and limited number of diagnostic traits. In this study, we focus on the *Pristimantis myersi* group, an assemblage of small rainfrogs distributed throughout the Andes of Ecuador and Colombia, whose external morphology is highly conserved, and its species diversity and evolutionary relationships largely unknown.

**Methods:**

We inferred a new phylogenetic hypothesis for the frog genus *Pristimantis*, including all available sequences of the mtDNA 16S rRNA, as well as new DNA sequences from 175 specimens. Our sampling included 19 of the 24 species currently recognized as part of the *Pristimantis myersi* group.

**Results:**

Our new evolutionary hypothesis recovered the *P. myersi* group as non-monophyletic and composed of 16 species. Therefore, we exclude *P. albujai, P. bicantus, P. sambalan,* and *P. nelsongalloi* in order to preserve the monophyly of the group. We discovered at least eight candidate species, most of them hidden under the names of *P. leoni, P. hectus, P. festae, P. gladiator*, and *P. ocreatus*.

**Discussion:**

Our results reveal the occurrence of a high level of cryptic diversity to the species level within the *P. myersi* group and highlight the need to redefine some of its species and reassess their conservation status. We suggest that the conservation status of six species within the group need to be re-evaluated because they exhibit smaller distributions than previously thought; these species are: *P. festae, P. gladiator, P. hectus, P. leoni, P. ocreatus*, and *P. pyrrhomerus*. Finally, given that the *Pristimantis myersi* group, as defined in this work, is monophyletic and morphologically diagnosable, and that *Trachyphrynus* is an available name for the clade containing *P. myersi*, we implement *Trachyphrynus* as a formal subgenus name for the *Pristimantis myersi* group.

## Introduction

*Pristimantis* Jiménez de la Espada, 1870, is the most diverse genus of terrestrial frogs, with 595 species described (Frost, 2023). It is distributed from Honduras to southern Brazil, but concentrates a substantial part of its richness in the Andes of Colombia, Ecuador, and Peru (Lynch & Duellman, 1997; Heinicke, Duellman & Hedges, 2007; Hedges, Duellman & Heinicke, 2008; Duellman & Lehr, 2009; García et al., 2012; Frost, 2023). The taxonomy of *Pristimantis* is challenging because of its high diversity, relatively low intraspecific phenotypic variation, and few external diagnostic morphological characters (Lynch & Duellman, 1997; Duellman & Lehr, 2009; Guayasamin et al., 2015). Recent studies have shown that *Pristimantis* species richness is underestimated in part by the existence of morphologically cryptic species (Elmer & Cannatella, 2009; Padial & De la Riva, 2009; Yánez-Muñoz et al., 2010; Hutter & Guayasamin, 2015; Ortega-Andrade et al., 2015; Rivera-Correa & Daza, 2016; González-Durán et al., 2017; Páez & Ron, 2019).

Conceptual advances in the definition of species (de Queiroz, 2007; Padial & De la Riva, 2010; Camargo & Sites, 2013), and the implementation of integrative approaches including the use of DNA sequences, morphological and behavioral data, has allowed more robust and objective species delimitations. However, the inference of phylogenetic relationships based on molecular data is still pending for most *Pristimantis* species, despite several recent efforts (Bickford et al., 2007; Heinicke, Duellman & Hedges, 2007; Hedges, Duellman & Heinicke, 2008; Padial et al., 2010; Padial, Grant & Frost, 2014; Rivera-Correa & Daza, 2016; González-Durán et al., 2017; Guayasamin, Arteaga & Hutter, 2018; Páez & Ron, 2019), which limits the ability to identify and delimit independent evolutionary lineages hidden within complexes of morphologically cryptic species.

Our study focused on the *Pristimantis myersi* group. This assemblage has been phenetically defined by the combination of the following character states: small body size (SVL <28 mm), robust bodies, relative narrow heads and short snouts, cranial crest absents, tympanum differentiated, vocal slits and vomerine teeth present (vocal slits only absent in *P. floridus*), limbs short to moderate long, finger I shorter than finger II, toe V slightly longer than toe III and not extending to the proximal edge of the distal subarticular tubercle of the toe IV, and digital discs narrow (expanded in *P. floridus*) and rounded (lanceolate in *P. hectus*, *P. lucidosignatus*, and *P. onorei*) (Lynch & Duellman, 1997; Hedges, Duellman & Heinicke, 2008; Rödder & Schmitz, 2009).

The species belonging to this group inhabit rainforest, montane forests, and paramos of Colombia and Ecuador (Hedges, Duellman & Heinicke, 2008; Padial, Grant & Frost, 2014; Rojas-Runjaic, Delgado & Guayasamin, 2014; Rojas-Runjaic & Guayasamin, 2015; González-Durán et al., 2017; Guayasamin, Arteaga & Hutter, 2018). The group, as currently defined, is composed of 24 species: *Pristimantis festae* (Peracca, 1904); *Pristimantis myersi* (Goin & Cochran, 1963); *Pristimantis celator* (Lynch, 1976a); *Pristimantis leoni* (Lynch, 1976b); *Pristimantis gladiator* (Lynch, 1976b); *Pristimantis pyrrhomerus* (Lynch, 1976b); *Pristimantis ocreatus* (Lynch, 1981a); *Pristimantis repens* (Lynch, 1984); *Pristimantis hectus* (Lynch & Burrowes, 1990); *Pristimantis verecundus* (Lynch & Burrowes, 1990); *Pristimantis scopaeus* (Lynch, Carranza & Robayo, 1996); *Pristimantis floridus* (Lynch & Duellman, 1997); *Pristimantis xeniolum* (Lynch, 2001); *Pristimantis jubatus* (García & Lynch, 2006); *Pristimantis bicantus* Guayasamin & Funk, 2009; *Pristimantis onorei* Rödder & Schmitz, 2009; *Pristimantis lucidosignatus* Rödder & Schmitz, 2009; *Pristimantis sirnigeli* Yánez-Muñoz et al., 2010; *Pristimantis munozi* Rojas-Runjaic, Delgado & Guayasamin, 2014; *Pristimantis mutabilis* Guayasamin et al., 2015; *Pristimantis sambalan* Brito, Batallas & Yánez-Muñoz, 2017; *Pristimantis albujai* Brito, Batallas & Yánez-Muñoz, 2017; *Pristimantis gralarias* Guayasamin, Arteaga & Hutter, 2018; and *Pristimantis nelsongalloi* Valencia et al., 2019. Of these, 20 are found in Ecuador, between 900 and 4,150 masl (Frost, 2023).

As occur in other co-generic clusters, the *Pristimantis myersi* group is characterized by a remarkable paucity of discrete phenotypic characters useful to diagnosing the group. At the same time, some species in this group may exhibit striking intraspecific phenotypic variation in color pattern and skin texture (*e.g.*, Arteaga et al., 2016; Rojas-Runjaic, Delgado & Guayasamin, 2014; Guayasamin et al., 2015). The combination of such phenomena represents a major source of taxonomic confusion; and the occurrence of instances of sympatry among some species of this group (*e.g.*, Yánez-Muñoz et al., 2010; Rojas-Runjaic, Delgado & Guayasamin, 2014; Guayasamin et al., 2008) adds complexity to the system. In consequence, the occurrence of cryptic species (*i.e.,* more than one morphologically similar species erroneously classified under the same name), taxonomic synonyms (*i.e.,* two or more species names assigned to the same taxonomic species), and a very high percentage of species misidentifications in museum specimens is likely for the *P. myersi* group. These issues have been already highlighted by Yánez-Muñoz et al. (2010), who suggested that *P. onorei* and *P. lucidosignatus* may be junior synonyms of *P. pyrrhomerus* and *P. floridus*, respectively; and are also exemplified by the finding of the first record of *P. myersi* from museum specimens long misidentified as *P. festae* and *P. ocreatus* (Rojas-Runjaic & Guayasamin, 2015). Discovering the phylogenetic relationships of the *Pristimantis myersi* group and clarifying its taxonomic uncertainties is important, not only to improve our knowledge on its current species richness and to make adequate ecologic and biogeographic interpretations, but also for conservation purposes (Ortega-Andrade et al., 2021), such as re-evaluating the conservation status of already named species whose known extent of occurrence is altered as a consequence of their taxonomic redefinition, evaluating without further delay the status of those still undescribed, and to adequately guide conservation actions for all of them.

Our study aimed to assess the phylogeny and species diversity of the *Pristimantis myersi* group from a molecular perspective, and through a notably expanded taxon and geographic sampling, especially in Ecuador. This phylogenetic information allowed us to: (i) infer the evolutionary relationships between species of the group, (ii) redefine the species content of the group to render it monophyletic, and (iii) assess species diversity and species limits.

## Materials & Methods

### Ethics statement

We follow the guidelines for use of live amphibians and reptiles in field research from Beaupre et al. (2004).

### Taxon sampling

We follow the taxonomy of Hedges, Duellman & Heinicke (2008) and Padial, Grant & Frost (2014) regarding family, genus, and arrangement of species groups. New mitochondrial DNA sequences from 175 specimens representing 31 species, were generated in the Laboratorio de Biología Evolutiva at Universidad San Francisco de Quito USFQ (LBE-USFQ), and the BioCamb’s molecular laboratory at Universidad Tecnológica Indoamérica (Appendix 1). Homologous sequences of some species in the *P. myersi* group available from previous studies as well as those of the outgroups, were downloaded from GenBank (http://www.ncbi.nlm.nih.gov/genbank/), 16 GenBank sequences required reidentification (Table 1). Voucher specimens for the new sequences are deposited at the Museo de la Universidad Tecnológica Indoamérica, Quito (MZUTI), Museo de Zoología de la Universidad San Francisco de Quito (ZSFQ), Museo de Zoología de la Pontificia Universidad Católica de Ecuador, Quito (QCAZ), and División de Herpetología (DHMECN) of the Instituto Nacional de Biodiversidad (INABIO). Other acronyms associated with terminals included in our phylogeny are JMG (Juan Manuel Guayasamin, field series, at USFQ), KU (University of Kansas, United States of America), and UVC (Universidad del Valle, Colombia).

Our DNA sequence data includes 19 species currently assigned to the *Pristimantis myersi* group (*P. albujai*, *P. bicantus*, *P. celator*, *P. festae, P. gladiator*, *P. gralarias*, *P. hectus*, *P. jubatus*, *P. leoni*, *P. lucidosignatus*, *P. munozi*, *P. mutabilis*, *P. nelsongalloi*, *P. ocreatus*, *P. onorei*, *P. pyrrhomerus*, *P. sambalan*, *P. sirnigeli*, and *P. verecundus*), which represent coverage of 79% of the group (Appendix 1). Our first phylogenetic analysis included all available sequences of *Pristimantis* (760 terminals); the inferred tree allowed us to restrict the *P. myersi* group and, at the same time, to identify species that were erroneously placed in the group. In a second inference, we included only species of the monophyletic *P. myersi* group and closely related clades (219 terminals; Appendix 1). We included 10 species of several craugastorid genera as outgroups (*Craugastor daryi*, *C. longirostris*, *Diasporus hylaeformis*, *D. vocator*, *Lynchius flavomaculatus*, *L. nebulanastes*, *Oreobates cruralis*, *O. saxatilis*, *Phrynopus auriculatus*, and *P. bracki*) (Pinto-Sánchez et al., 2012), and used *Agalychnis callidryas* to root the trees. Due logistic limitations we were unable to obtain samples of three species of the *Pristimantis myersi* group whose geographic distributions are restricted to Colombia (*i.e., P. repens*, *P. scopaeus*, and *P. xeniolum*). We also failed to include samples of *P. floridus* in our study, as this species has not been found in recent years.

**Table 1:** Species names of GenBank sequences re-identified in this study.

**Terminal name**	**Original name (GenBank code)**
*Pristimantis festae*	*Pristimantis trepidotus* (EF493515)
*Pristimantis ocreatus*	*Pristimantis thymelensis* (JX564889)
*Pristimantis* sp. 1	*Pristimantis hectus* (JN104680)
*Pristimantis* sp. 1	*Pristimantis hectus* (JN371037)
*Pristimantis* sp. 2	*Pristimantis verecundus* (EF493686)
*Pristimantis* sp. 10	*Pristimantis verecundus* (KM675445)
*Pristimantis* sp. 10	*Pristimantis verecundus* (KM675446)
*Pristimantis* sp. 10	*Pristimantis verecundus* (KM675447)
*Pristimantis* sp. 10	*Pristimantis verecundus* (KM675448)
*Pristimantis* sp. 10	*Pristimantis verecundus* (KM675464)
*Pristimantis* sp. 10	*Pristimantis verecundus* (KM675465)
*Pristimantis* sp. 10	*Pristimantis verecundus* (KM675466)
*Pristimantis* sp. 10	*Pristimantis verecundus* (KM675467)
*Pristimantis* sp. 13	*Pristimantis pyrrhomerus* (EF493683)
*Pristimantis* sp. 18	*Pristimantis leoni* (EF493684)
*Pristimantis* sp. 18	*Pristimantis librarius* (MH516183)

### DNA extraction and PCR amplification for 16S rRNA gene

For DNA extraction, we followed the guanidine thiocyanate protocol designed by Peñafiel et al. (2019). For the Polymerase Chain Reaction (PCR) amplification of the 16S rRNA gene fragment, we followed the protocol designed by Guayasamin et al. (2017). For DNA amplification, we used the primers 16SC (GTRGGCCTAAAGCAGCCAC) and 16Sbr-H (5′-CCG GTC TGA ACT CAG ACG T-3′) designed by Darst & Cannatella (2004) and Palumbi et al. (1991), respectively. Each PCR reaction contained a final concentration of 1.5 mM MgCl2, 0.5 mM dNTP, 0.25 U/µL of Taq (Invitrogen) DNA polymerase, and 0.2 µM of each primer, in a total volume of 25 µL. The PCR protocol included an initial denaturation step of 4 min at 94 °C; followed by 1 min at 94 °C, 30 s at 57 °C, and 2 min at 72 °C, for 30 cycles, and a final extension of 8 min at 72 °C. The amplicons obtained were visualized by electrophoresis with a 2% agarose gel. The amplified samples were cleaned with ExoSAP, and cycle sequencing reactions were performed by Macrogen Sequencing Labs (Macrogen Inc., Korea). All fragments were sequenced in both forward and reverse directions.

The utility of mitochondrial genes, especially 16S, to uncover population structure and cryptic species has a long history in amphibian studies and has been the most widely used to delimit species (Fouquet et al., 2007; Funk, Caminer & Ron, 2012; Cryer et al., 2019; Páez & Ron, 2019; Guayasamin et al., 2020; Sánchez-Nivicela et al., 2021); moreover, because of the longer coalescence times of nuclear genes (approximately four times that of mtDNA), studies on closely related taxa heavily rely on fast-evolving genes (Avise, 2000; Vences et al., 2005; Zink & Barrowclough, 2008).

### Sequence editing and phylogenetic analyses

All chromatogram sequences were fully inspected, assembled, compared against their reverse complements to detect errors, and manually edited using Geneious Pro 5.4.6 (Genematters Ltd.). We performed BLAST queries (https://blast.ncbi.nlm.nih.gov/Blast.cgi) for all the new sequences to verify their identity and to discard contaminations or mislabeling errors. GenBank accession codes of the generated sequences for the *Pristimantis myersi* group are in Appendix 1.

We performed the alignment with the online program MAFFT v7 (Katoh & Standley, 2013) under G-INS-I strategy (available at: https://mafft.cbrc.jp/alignment/server/index.html), and visualized it in Mesquite v3.6 (Maddison & Maddison, 2019). Uncorrected pairwise distances were calculated in MEGA7 (Kumar, Stecher & Tamura, 2016). Phylogenies were performed using Maximum Likelihood (ML) and Bayesian Inference (BI) methods. To obtain the best nucleotide substitution model, we used Model-Finder under the Bayesian information criterion (BIC) (Kalyaanamoorthy et al., 2017). Phylogenetic inference was made using ten replicates under the Maximun Likelihood (ML) criterium in IQ-Tree v2.1.3., which simultaneously finds the topology, and branch lengths that maximize the log-likelihood (Nguyen et al., 2015). Branch support was assessed from 100,000 ultrafast bootstraps (UFBoot2) replicates (Hoang et al., 2018; Minh, Nguyen & Haeseler, 2013), and SH-like approximate likelihood ratio test (Anisimova & Gascuel, 2006). To decrease the risk or overestimation branch support produced by UFBoot2, we implemented UFBoot optimization by nearest neighbor interchange search.

We produced two phylogenetic results using this methods. The first one using all available sequences of *Pristimantis* (742 terminals) and the second one using *P. myersi* group and closely related clades (215 terminals). For the first (742 terminals) we generated three phylogenetic trees using the ML described above and we made a topology test of them. The topology test was made using 1,000,000 replicates to analyze the Bootstrap Proportion using RELL approximation, Kishino-Hasegawa Test, Shimodaira-Hasegawa Test, Expected Likelihood Weights, and Approximately Unbiased (AU) Test (Kishino & Hasegawa, 1989; Kishino, Miyata & Hasegawa, 1990; Shimodaira & Hasegawa, 1999; Shimodaira, 2002; Strimmer & Rambaut, 2002), in IQ-Tree v2.1.3. All the topologies meet the 95% confidence set in all tests. For the second phylogeny, we generated 10 replicates using the ML method described above. Also, we produced two phylogenetic models of ML using regular bootstraps (Felsenstein, 1985); the branch support was obtained using 100 and 200 bootstraps, in IQ-Tree (Nguyen et al., 2015). The topology test was run as described in the previous analysis, and the selected topology meets the 95% confidence set in all tests. Bayesian inferences model were performed in MrBayes 3.2.7a software (Ronquist et al., 2012). We conducted four parallel runs of Monte Carlo Markov Chain (MCMC) for 10,000,000 generations, with sampling every 1,000 iterations and burning of 25% to estimate the Bayesian tree and Bayesian Posterior Probabilities (BPP). GTR+G+I was used as evolutionary model; this was selected using MrModelTest. Stationarity was assessed by examining the standard deviation of split frequencies and by plotting the –lnL per generation using Tracer 1.5.0 (Rambaut & Drummond, 2009). Finally, all trees generated were visualized in iTol v5 (Letunic & Bork, 2021) and edited in Adobe Illustrator 15.0.0 (Adobe Systems Inc.).

### Species concept and candidate species

We follow the evolutionary species concept of Simpson (1961) as modified by Wiley (1978), which define a species as a lineage of ancestral descendant populations that maintains its identity from other such lineages and that has its own evolutionary tendencies and historical fate. These lineages can be operationally defined by contingent properties (*e.g.*, fixed phenotypic traits, reproductive isolation, and reciprocal monophyly) that allow the discovery of their particular evolutionary trajectory (de Queiroz, 2007; Padial & De la Riva, 2010). Here, we delimited species and candidate species mainly based on molecular data and combining two approaches: monophyly verified by phylogenetic trees, and genetic distances (a non-tree based method).

Following Vieites et al. (2009) and Padial et al. (2010) we classified candidate species in three categories as follows: (1) Confirmed Candidate Species (CCS): deep genealogical lineages which exhibits fixed phenotypic characteristics that are consistent with its genetic divergence in differentiating them from other lineages, and that can be considered good species following standards of divergence for the group to which they belong, but that have not yet been formally described and named; (2) Unconfirmed Candidate Species (UCC): clades sister to nominal species, exhibiting relevant genetic divergence with respect to them (in this case we consider relevant distances ≥ 2%), but without further information on other lines of evidence (*e.g.*, bioacoustics, morphology); and (3) Deep Conspecific Lineages (DCL): lineages exhibiting intraspecific divergence values above the typical threshold observed for conspecific populations of related species, but where other lines of evidence indicate that they do not differ at the species level. In these cases, genetic divergence might be correlated to geographic distance.

Genetic distances are a powerful tool for identifying and discovering species. When gene flow between populations is restricted, genetic distances should increase (Nei, 1972; Janzen, 2004). Thus, genetic distances between populations of the same species should be smaller than between different species. To identify candidate species, we set a 2% threshold of uncorrected distances for the gene 16S, because there is empirical evidence indicating that sister species in *Pristimantis* usually exhibit such genetic distance or greater (*e.g.*, Padial & De la Riva, 2009; Ortega-Andrade et al., 2015) and other Neotropical frogs often differ by genetic distances <3% (*e.g.*, Coloma et al., 2012; Funk, Caminer & Ron, 2012; Jungfer et al., 2013; Caminer & Ron, 2014; Blotto et al., 2021; Escalona et al., 2021).

## Results

### Taxon and geographic sampling

We were able to include samples of topotypic specimens (or from specimens collected near the type localities of their respective species) of all (but *Pristimantis floridus* and *P. myersi*) the already named Ecuadorian species of the *Pristimantis myersi* group, in our phylogenetic analyses. This represents a coverage of 79% of the species currently included in this species group. In addition, we included a number of specimens corresponding to undetermined (and presumably new) species belonging to the *P. myersi* group.

### Phylogenetic relationships among species of the *Pristimantis myersi* group and its closely related clades

**Figure 1:**
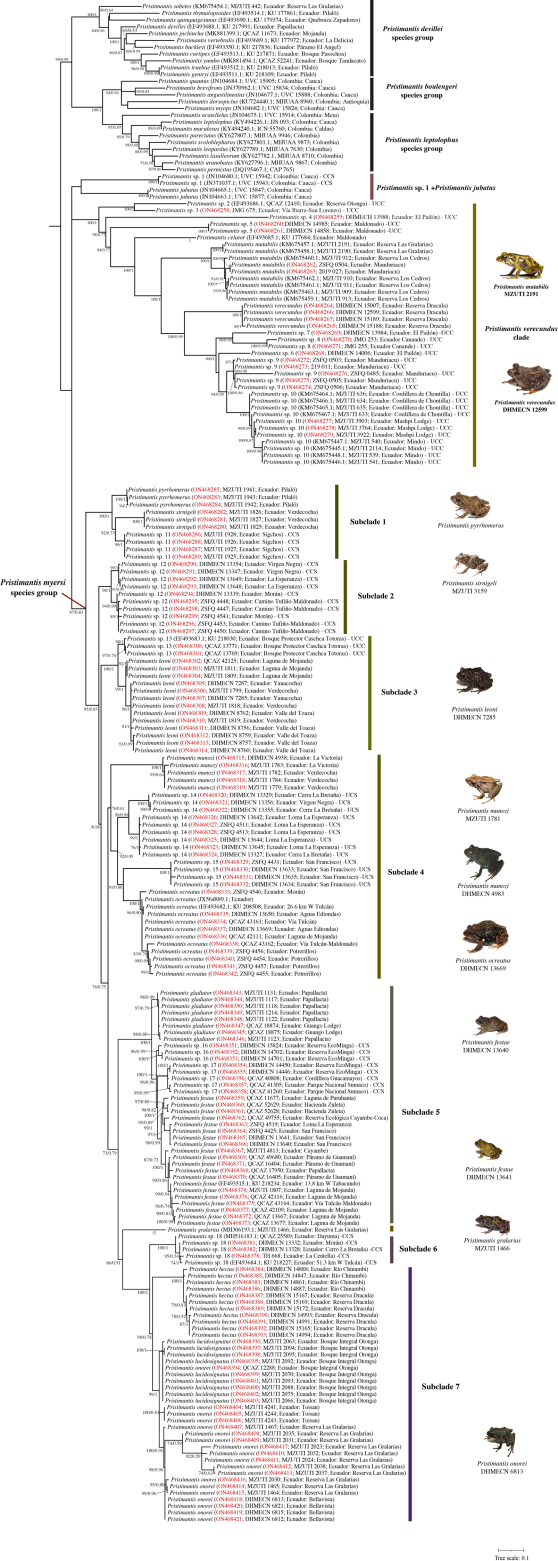
Maximum likelihood tree of the *Pristimantis myersi* species group and close relatives based on the mitochondrial gene 16S. Node support is expressed in Bootstrap values (%), followed by Bayesian posterior probabilities; missing values indicate support below 70% (bootstrap) or 0.5 (posterior probability). Each terminal includes the following information: species name, GenBank code, voucher number, and locality. Outgroups are not shown. Codes in red indicate new sequences. Abbreviations: CCS = confirmed candidate species, and UCS = unconfirmed candidate species. Photographs of *P. ocreatus* and *P. festae* was taken by Diego Batallas-Revelo; photographs of *P. verecundus*, *P. leoni*, *P. munozi* and *P. onorei* was taken by Mario Yánez Muñoz. The photographs of *P. mutabilis*, *P. pyrrhomerus*, *P. sirnigeli*, and *P. galarias* are from Juan M. Guayasamin.

The optimal nucleotide substitution model for our first dataset (742 terminals) according to Model-Finder (lnL = 50343.3980; BIC = 112673.3001) was TlM2+F+R7. This first topology (Supplemental File 1) allowed us to exclude species previously assigned to the *Pristimantis myersi* group (see below). For the second dataset (215 terminals) the optimal nucleotide substitution model according to Model-Finder (lnL = −10135.4133; BIC = 23218.6410) was TlM2+F+R3, and our optimal ML topology is shown in Fig. 1 (lnL = −10091.1778). For Bayesian Inference analyses, MrModelTest selected GTR+I+G as the best substitution model (lnL = −53947.9102; AIC = 107915.8203). Maximum Likelihood and Bayesian topologies obtained from the second dataset are largely congruent. In general, Bayesian tree showed higher nodal support and lower number of collapsed nodes than the ML tree. Since no relevant incongruences were found, we present only the Maximun Likelihood tree of the second dataset, including support values for each node obtained from both ultrafast bootstraps of ML and Bayesian posterior probability (*i.e.,* UFB/BPP) (Fig. 1). This tree is, in general, well-resolved, with most of the species previously assigned to the *Pristimantis myersi* group recovered as part of a well-supported clade (87%/0.83).

The clade of *Pristimantis myersi*, is grouped into a polytomy with other two strongly supported clades, one of them containing *P. celator*, *P. mutabilis*, *P. verecundus,* and several unnamed lineages (hereafter, *P. verecundus* clade; support = 100%/1; interspecific genetic distances: 2.6–16.3%), and the other (84%/1) composed of *P. jubatus* and a deeply divergent CCS (*Pristimantis* sp. 1); uncorrected p-distance between these two species was 8.2–8.3%. The successive sister clade (99%/0.99) to that described above, is composed of three monophyletic subclades corresponding to the *P. devillei*, *P. boulengeri*, and *P. leptolophus* species groups (Fig. 1).

From the 24 species previously assigned to the *Pristimantis myersi* group and sampled in our phylogeny, four (*P. albujai, P. bicantus, P. nelsongalloi,* and *P. sambalan*) were not recovered as part of that group. Contrary to expectations, *P. albujai* was inferred as sister to *P. cf. mendax*; the other three species were grouped in a clade (94%) that also includes *P. caprifer* and a sister clade that includes *P. acuminatus* and *P. eriphus*. Within this clade, the position of *P. nelsongalloi* renders *P. bicantus* paraphyletic (Fig. S1).

The *Pristimantis verecundus* clade (Fig. 1) is represented in our phylogeny by its nominal species (from Dracula Reserve, Carchi province, Ecuador), *P. celator* (Carchi province, Ecuador), *P. mutabilis* (Pichincha province, Ecuador), and nine Unconfirmed Candidate Species (UCC), namely: *Pristimantis* sp. 2 (Otonga, Cotopaxi province, Ecuador), *Pristimantis* sp. 3 (San Lorenzo road, Carchi province, Ecuador), *Pristimantis* sp. 4 (Dracula Reserve, Carchi province, Ecuador), *Pristimantis* sp. 5 (Maldonado-Chinambí road, Carchi province, Ecuador), *Pristimantis* sp. 6 (Dracula Reserve, Carchi province, Ecuador), *Pristimantis* sp. 7 (Dracula Reserve, Carchi province, Ecuador), *Pristimantis* sp. 8 (Canandé Reserve, Esmeraldas province, Ecuador) *Pristimantis* sp. 9 (Manduriacu Reserve, Pichincha province, Ecuador), and *Pristimantis* sp. 10 (Mindo, Mashpi, and Chontilla, Pichincha province, Ecuador). Interspecific uncorrected 16S p-distances within this group range between 2.6 and 16.3% (Table S2).

**Figure 2:**
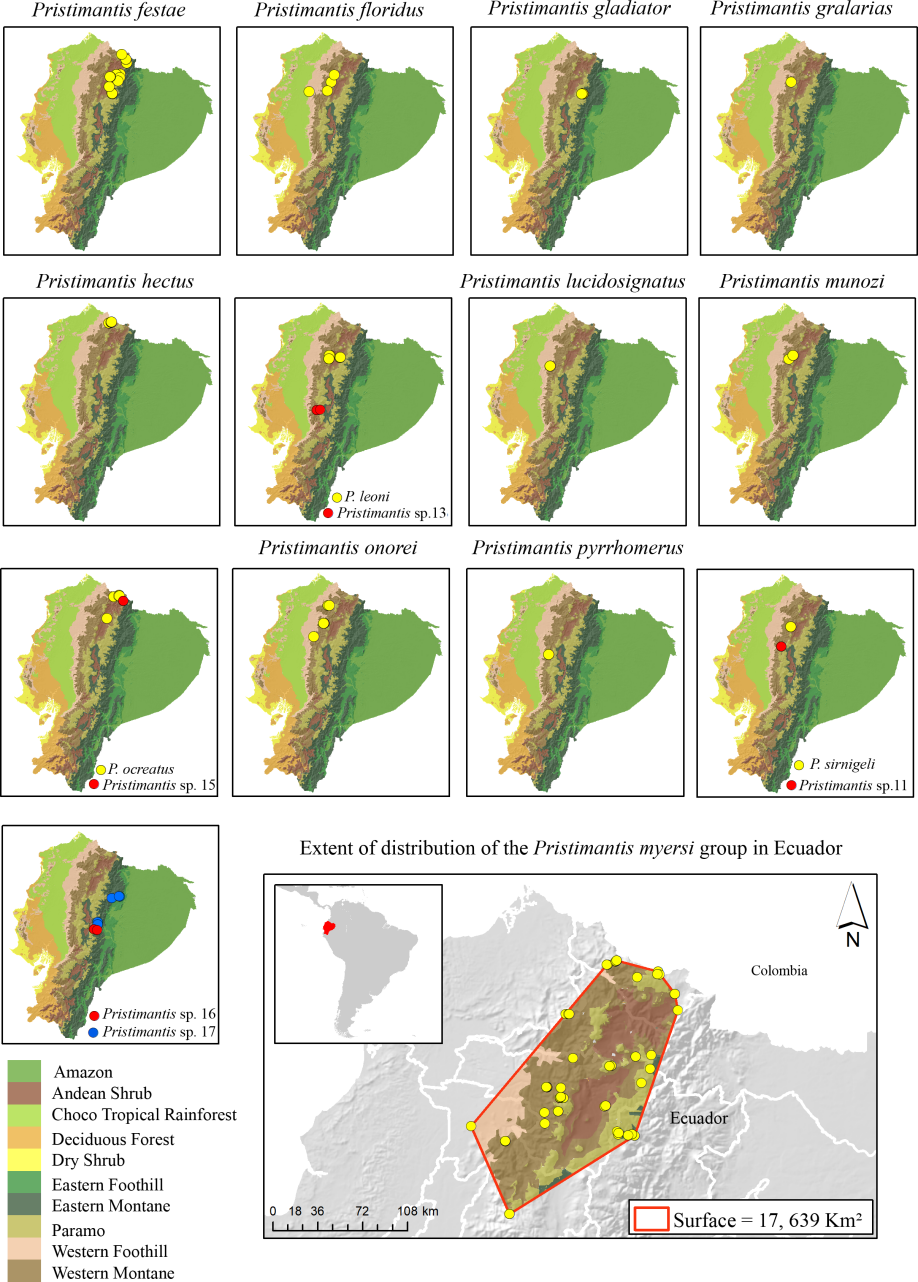
Distribution of the species in the *Pristimantis myersi* group (12 named species + five new candidate species) in the biogeographic regions of Ecuador. Land cover: Modified from Ron, Guayasamin & Menéndez-Guerrero (2011).

Within the *Pristimantis myersi* group we identified at least 18 independent and well-supported lineages corresponding to ten named species and at least eight candidate species (Fig. 2). These species-level lineages are arranged in seven major subclades. Below we describe the species composition, evolutionary relationships, and interspecific genetic divergence within each of them.

*Subclade 1* (Fig. 1): A strongly-supported clade (100%/1) composed of three lineages, namely: *P. pyrrhomerus*, *P. sirnigeli*, and a CCS (*Pristimantis* sp. 11). *Pristimantis pyrrhomerus* is represented by topotypes (from ca. 2 km E of Pilaló, Cotopaxi province, Ecuador) and it is sister to the other two lineages. In turn, *P. sirnigeli* it is represented by topotypes from Reserva Ecológica Verdecocha, Pichincha province, Ecuador and was recovered as sister to *Pristimantis* sp. 11 (from Sigchos, Cotopaxi province, Ecuador), a deeply divergent, geographically distant and morphologically distinguishable lineage. Genetic *p*-distances between *P. sirnigeli* and their two successive sisters, range between 2.7 and 3.7%, whereas p-distance between *P. pyrrhomerus* and *Pristimantis* sp. 11 was 2.2–2.3% (Table S3).

*Subclade 2* (Fig. 1): A well-supported clade (98%/1) only composed of *Pristimantis* sp. 12, a CCS. All the specimens grouped in this subclade come from four different localities in Carchi province, Ecuador. The species exhibit genetic structure (although small) apparently related to geographic distance. Intraspecific genetic distances for this lineage do not surpass 0.8% (Table S4).

*Subclade 3* (Fig. 1): A fully-supported clade (100%/1) composed of two lineages, namely: *Pristimantis* sp. 13 (UCC) from Bosque Protector Cashca Totoras (Bolívar province, Ecuador), and *P. leoni*. The latter is represented by specimens from six localities along its entire known distribution on northern Ecuador, including the type locality (Nudo de Mojanda). *Pristimantis* sp. 13 and *P. leoni*, are reciprocally monophyletic and their genetic divergence ranges between 1.3–2.2% (Table S5).

*Subclade 4* (Fig. 1): A supported clade (76%/0.61) composed of four species: *Pristimantis munozi*, *P. ocreatus*, and two morphologically cryptic UCC. *Pristimantis ocreatus* is represented by specimens from at least six different locations along northern Ecuador, including its type locality (Tufiño, Carchi province, Ecuador) and exhibits genetic structure. It is sister to *Pristimantis* sp. 15, a deeply divergent UCC from Carchi province in northern Ecuador. *Pristimantis* sp. 14 is the successive sister of *P. ocreatus* and *Pristimantis* sp. 15, it is represented by specimens from three localities, also from Carchi province in northern Ecuador, and shows genetic structure. Finally, *Pristimantis munozi*, which is external to all other species in this subclade, is represented by type specimens from Verdecocha and an additional specimen from La Victoria, both localities are on the western slope of Pichincha volcano (Pichincha province, Ecuador). Uncorrected 16S p-distances among the species of this subclade ranges between 2.9 and 8.4% (Table S6). The sequence JX564889 (*P. thymelensis*) obtained from GenBank was recovered nested within *P. ocreatus* and shows none or minimal genetic divergent (0.0–0.8%) with other samples of this last species.

*Subclade 5* (Fig. 1): A fully-supported clade (100%/1) formed by four lineages: *Pristimantis gladiator*, *P. festae*, and two CCS (*Pristimantis* sp. 16 and *Pristimantis* sp. 17). *Pristimantis festae* is represented by specimens of at least ten localities along northern Ecuador (Carchi, Imbabura, Pichincha, and Napo provinces), including its type locality (Papallacta, Napo province), shows genetic structure, with intraspecific p-distances between 0.0–1.9%. It is slightly divergent of *Pristimantis* sp. 17, its sister species. *Pristimantis* sp. 17 in turn is represented by specimens from three localities on the eastern slopes of the Ecuadorian Andes. *Pristimantis* sp. 16 is the successive sister species of these, is sympatric with *Pristimantis* sp. 17 at EcoMinga Reserve (Tungurahua province) and both are virtually indistinguishable from morphology. Finally, *Pristimantis gladiator*, which includes individuals from the type locality (Papallacta-Cuyuja, eastern versant of the Ecuadorian Andes) and surroundings, is the most external species and sister to all other lineages in the subclade. Interspecific genetic distances between all the lineages within this subclade ranges between 1.5–2.9% (Table S7).

*Pristimantis gralarias* (Fig. 1) is not treated as part of any subclade; it is sister to a large clade comprising the subclades 6 to 8. Uncorrected 16S p-distances between *P. gralarias* and all the other species in its sister clade are higher than 3.6%.

*Subclade 6* (Fig. 1): A fully-supported clade (100%/1) only composed of a single CCS (*Pristimantis* sp. 18), from five localities in Orellana and Carchi provinces, Ecuador. Genetic distances between all the six terminals in this subclade range between 0.0 and 7.1% (Table S8).

Subclade 7 (Fig. 1): A well-supported clade (99%/1) composed of three species: *Pristimantis hectus*, *P. lucidosignatus*, and *P. onorei*. *Pristimantis hectus* is only represented by specimens from two different localities, distanced ca. 100 km from the type locality (Reserva La Planada, Colombia). It is sister to the group composed by the other two aforementioned species. *Pristimantis onorei* includes samples from four different localities at Pichincha (Las Gralarias and Bellavista reserves), Imbabura (Toisán), and Cotopaxi provinces (Otonga reserve, type locality), in Ecuador. Topotypic specimens morphologically attributable to *P. lucidosignatus* (Otonga reserve) were recovered intermixed and forming a polytomy with sympatric topotypic specimens of *P. onorei*, rendering the latter paraphyletic. Genetic distances between sympatric and topotypic specimens of *P. lucidosignatus* and *P. onorei* ranges between 0.0–0.3%; however, these distances increase to 0.9–2.9% between *P. onorei* from Toisán and other localities (Table S9). Finally, uncorrected p-distances between *P. lucidosignatus* + *P. onorei* and *P. hectus* were 2.9–6.4% (Table S9).

## Discussion

### Monophyly, phylogenetic relationships, and redefinition of the *Pristimantis myersi* group

Previous studies on the phylogenetic relationships of the genus *Pristimantis* inferred the *P. myersi* group—sensu Lynch & Duellman (1997); Hedges, Duellman & Heinicke (2008)—as monophyletic (Heinicke, Duellman & Hedges, 2007; Hedges, Duellman & Heinicke, 2008; Pyron & Wiens, 2011; Pinto-Sánchez et al., 2012; Guayasamin et al., 2015; González-Durán et al., 2017; Guayasamin, Arteaga & Hutter, 2018); however, in all of these studies the *P. myersi* species group was represented by a very limited taxon sampling, which included up to five species (*P. festae*, *P. gralarias*, *P. ocreatus*, and two unnamed species) which barely represent 13% of the named species currently assigned to the group. Sequences originally attributed to *P. pyrrhomerus* (KU 218030; GenBank accession number EF493683) and *P. leoni* (KU 218227; EF493684) by Heinicke, Duellman & Hedges (2007) correspond to two undescribed species (*Pristimantis* sp. 13 and *P*. sp. 18, respectively; see Fig. 1), whereas sequences referred as *P. myersi* by Guayasamin, Arteaga & Hutter (2018) (GenBank accession numbers: JX564889 and AY326009), originally referred as *P. thymelensis* by Darst & Cannatella (2004), were recovered as part of *P. ocreatus* in our phylogeny (see Fig. 1).

*Pristimantis bicantus*, *P. albujai*, *P. sambalan*, and *P. nelsongalloi* were all assigned to the *P. myersi* group in their original descriptions, solely on the basis of its overall similarity in external morphology (Guayasamin & Funk, 2009; Brito, Batallas & Yánez-Muñoz, 2017; Valencia et al., 2019); however, our phylogeny rejects these hypotheses, indicating that such morphological similarities may be better explained as evolutionary convergence of phenotypes among phylogenetically distant species. This phenomenon appears to be relatively common in Craugastoridae, where several phenetic groups have been shown to be non-monophyletic (Hedges, Duellman & Heinicke, 2008; Padial, Grant & Frost, 2014). Consequently, and in order to promote a supraspecific taxonomy based in natural groups (*i.e.,* groups including a single common ancestral and all its descendants), we exclude *P. albujai*, *P. bicantus*, *P. sambalan*, and *P. nelsongalloi* from the *P. myersi* group. This action renders the *P. myersi* group monophyletic.

*Pristimantis albujai* appears in our topology closely related to *P*. cf. *mendax*, which also is part of the *P. galdi* group (Padial, Grant & Frost, 2014) according the redefinition proposed by Targino (2016) in its unpublished thesis. In contrast, Zumel, Buckley & Ron (2021), recovered *P. albujai* as part of the *P. trachyblepharis* species group, which is not closely related to the *P. galdi* group. Therefore, we cannot corroborate the allocation of *P. albujai* in the *P. trachyblepharis* group as proposed by Zumel, Buckley & Ron (2021). In turn, *Pristimantis bicantus*, *P. nelsongalloi*, and *P. sambalan* are all grouped in a well-supported clade in which is nested *P. caprifer*. The latter was relegated as unassigned to species group by Padial, Grant & Frost (2014) but later treated by Targino (2016) as part of her newly defined *P. euphronides* species group. Following Targino (2016), we suggest assigning *P. bicantus*, *P. nelsongalloi*, and *P. sambalan* as part of *P. euphronides* species group.

Based on an alleged congruence between morphological similarity and molecular-based topologies, Guayasamin, Arteaga & Hutter (2018) proposed a more inclusive redefinition of the *P. myersi* group, which encompassed its two successive sister clades, composed of *P. verecundus*, *P. mutabilis*, *P. celator*, *P. jubatus*, and several unnamed species closely related to these four species. Previously, Targino (2016) had also re-defined the *P. myersi* group in the same way, but only based in topology, because no unambiguously optimized phenotypic synapomorphy was recovered for that group. As far as we noted, these newly included species phenotypically differ from the species in the original *P. myersi* group at least in having digital discs expanded (weakly expanded to unexpanded in the *P. myersi* group; except in *P. floridus*, but its phylogenetic position has not been corroborated by molecular data), and by having discoidal fold (absent in the *P. myersi* group). Moreover, *P. verecundus* and its related species are arboreal (Lynch & Burrowes, 1990) whereas species in the *P. myersi* group are terrestrial or perch close to the ground (Hedges, Duellman & Heinicke, 2008; Padial, Grant & Frost, 2014). *Pristimantis celator* and *P. jubatus* further differ in a number of characters (*i.e.,* absence of vocal slits) from all other species of the *P. myersi* group.

Taking into account that the expanded definition of the *Pristimantis myersi* group proposed by Targino (2016) and Guayasamin, Arteaga & Hutter (2018) lacks unambiguously optimized phenotypic synapomorphies, reduces its morphological diagnosability by making most characters in its definition polymorphic, and even is not supported in our study (Fig. 1), we conservatively propose returning to the previous definition of the *P. myersi* group (*i.e.,* in our topology, the most inclusive clade containing *P. pyrrhomerus* and *P. onorei*, but not the clades of *P. verecundus* and *P. jubatus;* see Fig. 1), which is less inclusive but more phenotypically diagnosable.

Although herein excluded of the *Pristimantis myersi* group, we briefly discussed the species composition of *P. verecundus* and *P. jubatus* clades, as we discovered cryptic diversity to the species level within both. In the last clade, *Pristimantis* sp.1 (represented by the specimens UVC 15942 and 15043, from Cauca, Colombia) is sister and deeply divergent to *P. jubatus* (Fig. 1). It was originally sampled by Mendoza et al. (2015) and determined as *Pristimantis hectus*. We were able to examine a photo of one of these specimens in life and corroborate it is morphologically similar to *P. hectus* (including the characteristic lanceolate discs of that species). Nevertheless, this lineage is not phylogenetically related to our specimens of *P. hectus* which phenotypically matching with the species description and come from two localities about 45 km SW from La Planada (type locality). This lineage represents a typical case of cryptic species hidden by morphological convergence under another phylogenetically not related species (Patiño Ocampo, Duarte-Marín & Rivera-Correa, 2022).

Within the *Pristimantis verecundus* clade we discovered an astonishing cryptic diversity, with nine of the 12 independent evolutionary lineages that compose the clade (or 75%) corresponding to UCC. All of them were originally determined as *P. verecundus* based on their overall external morphology; however, our genetic evidence (*i.e.,* topology and uncorrected p-distances) indicates that they presumably correspond to different species. All the species within this clade diverges in 6.3–14.6% from our samples of *P. verecundus sensu stricto* from Carchi province (Ecuador) about 45 km SW of La Planada, Colombia (type locality of *P. verecundus*). Further studies integrating morphology and bioacoustic data to our molecular evidence, will be required in order to corroborate the species limits within this clade.

In the subclade 1, the position of *Pristimantis sirnigeli* as part of the same clade of *P. pyrrhomerus* corroborates the close relationship between these two species already suggested by Yánez-Muñoz et al. (2010) solely on the basis of external morphology. On the other hand, based on a preliminary examination, *Pristimantis* sp. 11 (from Sigchos) is morphologically indistinguishable from topotypes of *P. pyrrhomerus* (from Pilaló) and their calls sound very similar (F.J.M. Rojas-Runjaic, pers. obs.), by which we originally determined the specimens from Sigchos as an additional population of *P. pyrrhomerus*. However, in our phylogenetic hypothesis these two are not sister lineages; *Pristimantis* sp. 11 is sister to *P. sirnigeli*, and *P. pyrrhomerus* is the successive sister of these two, so that *P. sirnigeli* renders *P. pyrrhomerus* paraphyletic (Fig. 1). Two different scenarios in which the monophyly of the group is preserved, can explain this situation: a) they are three deeply divergent conspecific lineages and *P. sirnigeli* is a junior synonym of *P. pyrrhomerus*, or b) *Pristimantis* sp. 11 is a morphologically cryptic species indistinguishable from *P. pyrrhomerus* but not directly related to that. Considering that, *P. sirnigeli* is readily distinguishable from their two successive sister lineages by morphology (*e.g.*, *P. sirnigeli* has remarkably longer and thinner fingers, with digital discs more expanded than in *P. pyrrhomerus*, and dorsal skin much more tuberculate) and in their calls (F.J.M. Rojas-Runjaic, pers. obs.), and that genetic distances among these three lineages are greater than 2.2%, with *P. sirnigeli* being the most divergent (2.7–3.7%), we conclude that *Pristimantis* sp. 11 is in fact a morphologically cryptic CCS.

The subclade 3, included *Pristimantis* sp. 13, a CCS that has been sampled in all previous phylogenies of the *P. myersi* group. Despite this, its condition of as an undescribed species went unnoticed until now due it was erroneously determined as *P. pyrrhomerus* by Heinicke, Duellman & Hedges (2007), likely based on its similarity in external morphology. In the absence of sequences of *P. pyrrhomerus* of type locality (included by first time in this study), the error was perpetuated in subsequent phylogenies. Here we demonstrate that this lineage although seemingly similar to *P. pyrrhomerus* is not related to that. *Pristimantis* sp. 13 is the southern taxonomic replacement of its sister species *P. leoni*.

The subclade 4, includes *Pristimantis* sp. 14 and *Pristimantis* sp. 15. Although our genetic evidence (*i.e.,* monophyly and genetic distances between 2.9 and 6.8%) strongly support these two lineages as independent species from *P. ocreatus*, we were unable to further analyze their morphology and bioacoustics; hence, we conservatively referred them as UCC. Lynch (1981) suggested that *P. ocreatus* is closely related to *P. trepidotus* (= *P. festae*), based on their similarity in size, proportions, hands and feet morphology, and color patterns. However, our phylogenetic analyses demonstrates that *P. ocreatus* is not closely related to *P. festae*.

Within subclade 5, we discovered two CCS (*Pristimantis* sp. 16 and *P.* sp. 17). Although they are sympatric at EcoMinga Reserve and are virtually indistinguishable in terms of morphology, they are not sister species. *Pristimantis festae*, to whom *P.* sp. 17 and *P.* sp. 16 are successive sister species, proved to be a widely distributed species in paramo environments of northeastern Ecuador. Its relatively wide geographic distribution is reflected in the genetic structured exhibited in our topology; however, besides it, the species shows a relatively low intraspecific genetic divergence (not higher than 1.9%).

The specimen QCAZ 11677 was determined as *Pristimantis myersi* and recovered as sister to *P. festae* in the unpublished phylogeny of Rojas-Runjaic (2012). In addition, that specimen was also determined as *P. myersi* (based on morphology) and illustrated in Rojas-Runjaic & Guayasamin (2015). Nevertheless, we treated this specimen herein as part of *P. festae*, mainly based on its incipient genetic divergence in relation to *P. festae*. Although alternatively, we could follow previous authors in treating QCAZ 11677 as *P. myersi*, and consequently also the more inclusive group containing it and the samples from San Francisco (which would render *P. “myersi”* and *P. festae* reciprocally monophyletic), the resulting intraspecific genetic divergence in *P. “myersi”* (up to 1.5%) would be similar to the divergence between it and *P. festae* (0.8–1.9%). We recognize the existence of sister species of amphibians exhibiting very low genetic distances, but they typically are further supported by the congruence of other lines of evidence, such as bioacoustics, and morphometry (*e.g.*, Escalona et al., 2021). Given that we were unable to assess additional evidence on this issue, and faced with the impossibility of ruling out that the specimen QCAZ 11677 has been wrongly determined by Rojas-Runjaic & Guayasamin (2015), we conservatively referred it as part of *P. festae*.

Phylogenetic relationships among specimens of subclade 6, from five different localities (most of them in Carchi province, northern Ecuador) are not well resolved, and several of them exhibit remarkably long branches (likely due poor quality of the sequence TH 668). Despite this, the clade containing them is fully supported. We found high intra-specific genetic divergence reaching a variability up to 7%, even within the same population, similar studies that use mitochondrial DNA marker has been increasingly applied for evaluating the levels of genetic divergence, detecting barriers for gene flow that include Andean anurans (see Guarnizo, Amezquita & Bermingham, 2009; Rivera-Correa, Jimenez-Rivillas & Daza, 2017; Restrepo, Velasco & Daza, 2017; Rivera-Correa et al., 2022). We consider all of them conspecific and corresponding to *Pristimantis* sp. 18, an unnamed CCS. Within this clade is nested the specimen KU 218227, originally referred by Heinicke, Duellman & Hedges (2007) as *P. leoni*. Until now, this was the only available sequence for that species and consequently was included as such species in all subsequent phylogenies of the *P. myersi* group. Our topology, which is based in an exhaustive taxonomic and geographic sampling, reveals that the specimen KU 218227 is not related to *P. leoni* and actually correspond to *Pristimantis* sp. 18. Consequently, our study is also the first one to infer the phylogenetic position of *P. leoni* within the *P. myersi* group. We also highlight that the sequence of 16S available at GenBank of the specimen QCAZ 25589, first identified as *P. librarius* by Waddell et al. (2018) and presumably from an Amazonian locality, was recovered as part of the clade 6. It was inferred as sister to all the other five terminals of the clade 6, but barely diverges in 0.2% from three of them. Our evidence strongly suggest that this sample actually correspond to *Pristimantis* sp. 18 and likely comes from somewhere in the north of Carchi province. The Amazonian locality associated to this museum sample must be a mistake.

Subclade 7, groups *Pristimantis hectus*, *P. lucidosignatus*, and *P. onorei*. The type locality of *Pristimantis hectus* (La Planada) is located in the department of Nariño, southern Colombia (Lynch & Burrowes, 1990), ca. 45 km NE from the two localities sampled for this species in our study. There is not available molecular data from topotypes of *P. hectus* to compare with our specimens; however, we are confident that they are conspecific as their localities are nearby the type locality, and the specimens fully match with the morphological definition of *P. hectus*. However, based on the general pattern of geographic distribution exhibited by most of the species in the *P. myersi* group (*i.e.,* species with small extent of occurrence), we consider unlikely that the geographic distribution of *P. hectus* extends much further south to Ecuador. There are some previous records of *P. hectus* for Pichincha, Cotopaxi and Imbabura (Appendix 1). These specimens morphologically resemble to that species but they likely correspond to different species.

Our phylogeny recovered *P. onorei* as paraphyletic due the position of topotypes morphologically attributable to *P. lucidosignatus* (*i.e.,* bearing light markings on shanks) intermixed in a polytomy with topotypes of *P. onorei*. Thus, *P. lucidosignatus* might represent a junior synonym of *P. onorei*.

### Definition of the *Pristimantis myersi* group and formalization of the subgenus *Trachyphrynus*

So far, no unambiguous phenotypic synapomorphies are known for the *Pristimantis myersi* group (Hedges, Duellman & Heinicke, 2008; Taboada et al., 2013). However, based on the newly inferred species composition of the group derived from our phylogeny, we phenotypically redefine it as follows (modified from Lynch & Duellman, 1997; Hedges, Duellman & Heinicke, 2008): (1) small body size (SVL in females 15.8–34.6 mm; in males 12.6–20.5 mm); (2) short snout; (3) robust body; (4) Toe V longer than Toe III, Finger I shorter than II; (5) digital discs narrow (expanded in *P. floridus*); and (6) cranial crests absent. In addition, all species in the group are found on low vegetation (<150 cm above the ground), at ground level, or underground. The *Pristimantis myersi* group as redefined herein, now contains 16 named species, namely: *P. festae*, *P. floridus*, *P. gladiator*, *P. gralarias, P. hectus*, *P. leoni*, *P. lucidosignatus, P. munozi*, *P. myersi, P. ocreatus*, *P. onorei*, *P. pyrrhomerus*, *P. repens, P. scopaeus*, *P. sirnigeli*, and *P. xeniolum*; and at least eight candidate species identified in our phylogeny. Despite *P. repens, P. scopaeus*, *P. xeniolum,* and *P. floridus* were not included in our phylogeny, we opted by maintain them as part of the group but highlighting that the assessment of their phylogenetic positions is pending.

Species groups have been widely and largely used in the taxonomy of *Pristimantis* to organize and to make more manageable this hiperdiverse genus (Lynch & Duellman, 1997; Hedges, Duellman & Heinicke, 2008); however, this taxonomic category is informal as it is not ruled by the ICZN (1999). To solve this issue, some authors have proposed the implementation of subgenera to formally name and define monophyletic and diagnosable clades within *Pristimantis* (*e.g.*, Heinicke et al., 2018; Páez & Ron, 2019). *Trachyphrynus* (Goin & Cochran, 1963) was originally proposed as a genus name to allocate *Trachyphrynus myersi* (Goin & Cochran, 1963) but it was subsequently synonymized into *Eleutherodactylus* by Lynch (1968) and later into *Pristimantis* by Hedges, Duellman & Heinicke (2008). Considering that *P. myersi* is the nominal species of the homonymous species group, and that *Trachyphrynus* is an available name for the clade containing *P. myersi*, we implement *Trachyphrynus* as a formal subgenus name for the *Pristimantis myersi* group, as defined and delimited in this study.

### Speciation

Lynch & Duellman (1997) described three general patterns of speciation in Ecuadorian *Pristimantis*: latitudinal, altitudinal, and trans-Andean replacement. Species in the *P. myersi* group mainly follow a pattern of latitudinal and altitudinal speciation rather than trans-Andean. For example*, P. sirnigeli* is sister to *Pristimantis* sp. 11 (Fig. 1); both species are on the Pacific versant of the Andes, but at different elevations and latitudes (Appendix 1, Fig. 2); the two species might be geographically isolated by the Toachi/Jacuntama/Sarapuyo/Pilatón river basin. A similar pattern is found between *Pristimantis leoni* and *Pristimantis* sp. 13 (Fig. 1). *Pristimantis ocreatus* is sister to *Pristimantis* sp. 15 (Fig. 1); the two species inhabit similar elevation and are geographically close, but isolated by a valley (Fig. 2). Canyons and dry valleys are the most likely geographic elements that disrupt gene flow (*e.g.*, Lynch & Duellman, 1997; Coloma et al., 2012; Jungfer et al., 2013; Arteaga et al., 2016; Guayasamin et al., 2017; Guayasamin et al., 2020; Guayasamin et al., 2022; Yánez-Muñoz et al., 2018). For example, the Mira river valley restricts *P. hectus* to the north of Ecuador (Fig. 2); this basin is recognized as an important barrier for small vertebrates (Arteaga et al., 2016; Yánez-Muñoz et al., 2018). The canyon of the Pastaza River separates *P. gladiator*, *Pristimantis* sp. 16, and *Pristimantis* sp. 17 (Fig. 2). It seems that amphibian micro-endemism might mirror patterns of organisms already found in the same basin such as orchids and angiosperms (Jost, 2004; Jost & Shepard, 2017; Matsuda, 2018). There are also few examples of species that have reached new ecological zones (*e.g.*, *P. ocreatus* found as high as higher 4,150 m).

This pattern agrees with the hypothesis that long mountain ranges fragmented by narrow transverse valleys promote allopatric divergence by limiting contact among contiguous populations, also explaining the limited distribution of numerous Andean frogs (Remsem, 1984; Graves, 1988; Wollenberg et al., 2008; Hutter & Guayasamin, 2015; Guayasamin et al., 2020; Guayasamin et al., 2022; Burrowes et al., 2020; De la Riva, 2020). A scenario of mostly allopatric speciation could serve to explain the presence of cryptic species; since selective pressures (biotic and abiotic) are similar for allopatric species that occupy analogous environmental niches; thus, ancestral morphologies and behaviors may be retained (Peterson, Soberón & Sánchez-Cordero, 1999; Graham et al., 2004; Wiens & Graham, 2005).

Wiens (2004) posited that lack of variability in populations, natural selection, pleiotropic effects, and gene flow from the centers of populations to their peripheries can act together to stabilize allopatric populations about their ancestral niche. If these processes are indeed at play in diverging lineages, we might then expect allopatric sister species in the *P. myersi* group to retain their ancestral morphologies and behaviors, resulting in cryptic species. However, while morphological traits are often informative of ecology, this is not necessarily the case (Losos, 2008). In order to make such a judgement in the case of cryptic species of *Pristimantis*, more detailed investigations into the relationships between ecology and the common morphology and behaviors in cryptic species pairs need to be conducted. Furthermore, it is generally unknown whether the traits shared by cryptic species in the *P. myersi* group are functional. In the absence of such information, sexual selection, or non-adaptive and non-directly selective explanations such as pleiotropy are equally plausible explanations for the retention of ancestral morphologies in allopatric cryptic species pairs as similar selective pressures. We expect such avenues of research to be particularly informative in developing a better understanding of cryptic speciation in Neotropical anurans.

### Diversity of the *Pristimantis myersi* group in Ecuador

Numerous *Pristimantis* species remain undescribed because of the limited number of useful morphological traits for such a diverse genus. Thus, it should not surprise us that cryptic diversity is rampant when using alternative approaches, such as molecular phylogenies (Padial & De la Riva, 2009; Díaz, Hedges & Schmid, 2012; Padial et al., 2012; Fouquet et al., 2007; Kieswetter & Schneider, 2013; Hutter, Lambert & Wiens, 2017; Guayasamin et al., 2017; Guayasamin et al., 2022; Páez & Ron, 2019; Urgiles et al., 2019).

The higher species richness of the *Pristimantis myersi* group is concentrated in the montane forests of Ecuador. The group reaches its diversity peak in the western montane forest of the Andes, with nine named species (*P. festae*, *P. floridus*, *P. gralarias*, *P. hectus*, *P. leoni*, *P. lucidosignatus, P. munozi*, *P. pyrrhomerus*, *P. onorei*, and *P. sirnigeli*) in an altitudinal range from 1,589 to 3,487 m asl. Then, in the Paramo, with four named species (*P. festae*, *P. gladiator*, *P. myersi*, and *P. ocreatus*) in an altitudinal range from 3,853 to 4,068 m asl; and Andean shrub with two species (*P. festae* and *P. leoni*) in an altitudinal range from 2,834 to 2,901 m asl (Fig. 2 and Appendix 1).

Based on the results of this study, the number of species of the *Pristimantis myersi* group, as defined herein, would increase from 16 species to 23 (16 already recognized + 8 new candidate species), meaning that 34.8% of the known to date diversity of the *P. myersi* group is yet undescribed. Previous estimates of cryptic diversity on Neotropical amphibians’ range between 22–400%, but these studies are restricted to the Amazon region and are based on non-terraranan species (Fouquet et al., 2007; Funk, Caminer & Ron, 2012; Jungfer et al., 2013; Gehara et al., 2014; Rojas et al., 2018; Jaramillo et al., 2020). To date, no estimation of cryptic diversity for the Andean amphibians has been published. Our estimates, although restricted to a relatively small group, may apply to other Andean anurans with similar characteristics (*e.g.*, Terrarana).

### Impact on conservation

Clarifying taxonomic uncertainties is imperative not only to reveal true species richness within a group, but also for conservation purposes. Our study provides information that affects the conservation status of several species. Currently, the conservation status of five species of the *Pristimantis myersi* group have not been assessed under the criteria of the IUCN and Ortega-Andrade et al. (2021) (Table 2). Moreover, the extinction risk of several species requires a re-evaluation for the following reasons: (1) most species in the *P. myersi* group have geographic distributions that are actually smaller than currently recognized; (2) most species inhabit paramo and montane forest areas that are afflicted by severe anthropogenic factors (Menéndez-Guerrero & Graham, 2013); (3) several species (*e.g.*, *P. festae*, *P. leoni*) actually represent species complexes. Specifically, we consider that the conservation status of six species within the group needs to be re-evaluated because they exhibit smaller distributions than previously thought; these species are: *P. festae*, *P. gladiator*, *P. hectus*, *P. leoni*, *P. ocreatus*, and *P. pyrrhomerus* (see Table 2). Finally, if the lineages identified as candidate species are described, this would require assessing their conservation status and, hopefully, designating priority areas for their conservation.

**Table 2:** Species in the *Pristimantis myersi* group, with information on the distribution and conservation status (IUCN and Ecuador’s Red List by Ortega-Andrade et al., 2021). Conservation status follows IUCN categories: Least concern (LC), Near Threatened (NT), Vulnerable (VU), Endangered (EN), No Evaluated (NE), and No information (–).

**Species**	**Distribution**	**IUCN**	Ortega-Andrade et al. (2021)	**Observations IUCN**	**Sources**
***P. festae***	On the eastern slope of the Ecuadorian Andes, Sucumbíos, Napo and Tungurahua provinces and near southern Colombia. This study: Carchi, Pichincha, Imbabura, Napo province, Ecuador.	EN	VU	Requires re-evaluation	Coloma et al. (2004a), Frost (2023), Peracca (1904) and Ortega-Andrade et al. (2021)
***P. floridus***	Western flank of the Andes of Ecuador, Cotopaxi, Imbabura, and Pichincha provinces. This study: NA	VU	VU	−	Frost (2023), Lynch & Duellman (1997), Lynch, Coloma & Ron (2004) and Ortega-Andrade et al. (2021)
***P. gladiator***	On the Amazonian slopes of the Andes Carchi, Napo and Imbabura provinces; adjacent Putumayo, Colombia. This study: Napo province Ecuador.	VU	VU	Requires re-evaluation	Frost (2023), Almeida et al. (2019), Lynch (1976c) and Ortega-Andrade et al. (2021)
***P. gralarias***	Known only from the type locality, Ecuador (Pichincha province). This study: Type locality, Pichincha province, Ecuador.	−	VU	Requires evaluation	Frost (2023), Guayasamin, Arteaga & Hutter (2018) and Ortega-Andrade et al. (2021)
***P. hectus***	Western slope of the Cordillera Occidental in the Department of Nariño, Colombia; northwestern Ecuador (Esmeraldas and Imbabura provinces). This study: Carchi province, Ecuador.	VU	EN	Requires re-evaluation	Frost (2023), Cepeda-Quilindo et al. (2019), Lynch & Burrowes (1990) and Ortega-Andrade et al. (2021)
***P. leoni***	Southern Colombia and Amazonian slopes of the Andes in northern Ecuador (Carchi, Imbabura, Pichincha, Sucumbíos and Santo Domingo de los Tsáchilas provinces). This study: Only in the Imbabura and Pichincha provinces, Ecuador.	LC	EN	Requires re-evaluation	Castro et al. (2010), Frost (2023), Lynch (1976c), Lynch (1976b), Lynch (1976a) and Ortega-Andrade et al. (2021)
***P. lucidosignatus***	Known only from, Cotopaxi Province, and Tandapi, Pichincha Province, Ecuador.This Study: Cotopaxi province, Ecuador.	NE	DD	−	Frost (2023), Rödder & Schmitz (2009) and Ortega-Andrade et al. (2021)
***P. munozi***	Known only from the type locality (Pichincha province, Ecuador). This study: Type locality, Pichincha province, Ecuador.	−	VU	Requires evaluation	Frost (2023), Rojas-Runjaic, Delgado & Guayasamin (2014) and Ortega-Andrade et al. (2021)
***P. myersi***	On the southern, Cordillera Central in Colombia and in the North of Ecuador, Sucumbíos province. This study: We excluded Imbabura province.	LC	VU	−	Frost (2023), Castro, Herrera & Lymch (2004), Goin & Cochran (1963) and Ortega-Andrade et al. (2021)
***P. ocreatus***	Ecuador, Carchi, Imbabura, Napo and Cotopaxi provinces. This study: Carchi and Imbabura province, Ecuador.	EN	EN	Requires re-evaluation	Coloma et al. (2004b), Frost (2023), Lynch (1981a), Lynch (1981b) and Ortega-Andrade et al. (2021)
***P. onorei***	Ecuador, Santo Domingo de los Tsáchilas, Pichincha and Cotopaxi provinces. This study: Cotopaxi, Imbabura and Pichincha province, Ecuador.	−	DD	Requires evaluation	Frost (2023), Rödder & Schmitz (2009) and Ortega-Andrade et al. (2021)
***P. pyrrhomerus***	Ecuador, Pichincha, Imbabura, Carchi, Cotopaxi and Bolívar provinces. This study: Only Cotopaxi province, Ecuador.	EN	CR	Requires re-evaluation	Coloma et al. (2004c), Frost (2023), Lynch (1976b) and Ortega-Andrade et al. (2021)
***P. repens***	Colombia, Pasto and La Cruz, Department of Nariño. This study: NA	EN	−	−	Frost (2023), Rojas et al. (2019d) and Lynch (1984)
***P. scopaeus***	Known only from the type locality, Colombia (Municipal of Cajamarca). This study: NA	LC	−	−	Frost (2023), Castro, Gonzales-Duran & Herrera (2019) and Lynch, Carranza & Robayo (1996)
***P. sirnigeli***	Ecuador, Pichincha and Imbabura provinces. This study: Pichincha province, Ecuador.	−	EN	Requires evaluation	Frost (2023), Yánez-Muñoz et al. (2010) and Ortega-Andrade et al. (2021)
***P. xeniolum***	Known only from the type locality, Colombia (Department of Valle del Cauca). This study: NA	VU	−	−	Frost (2023), Castro et al. (2019) and Lynch (2001)

## Conclusions

*Pristimantis myersi* group (for which the name *Trachyphrynus* is applicable as subgenus), after the exclusion of *P. albujai*, *P. bicantus*, *P. nelsongalloi*, *P. sambalan*, *P. jubatus*, and *P. verecundus* clade, is monophyletic. Our study substantially contributes to a better understanding of the species richness and evolutionary relationships within the group. Species in the *P. myersi* group mainly follow mostly a pattern of latitudinal replacement rather than altitudinal or trans-Andean, promoting allopatric divergence. A scenario of allopatric speciation also explains the presence of cryptic species, but investigations into the relationships between ecology and the traits shared by particular cryptic species pairs are necessary to validate this hypothesis. Moving forward, obtaining calls would assist in solving species boundaries, particularly among sympatric lineages. We recommended an exhaustive taxonomic review of the *Pristimantis myersi* species group, as well as a re-evaluation of the conservation status of each species given the data provided herein.

##  Supplemental Information

10.7717/peerj.14715/supp-1 Supplemental Information 1 Appendix Species names, group/clade, GenBank accession numbers, voucher numbers, coordinates and localities of *Pristimantis myersi* species group, *P. verecundus* clade, *P. devillei*, *P. boulengeri* and *P. leptolophus* species groups.

10.7717/peerj.14715/supp-2 Supplemental Information 2 Part of maximum likelihood tree of DNA sequences of the mitochondrial gene 16S Node support is expressed in Bootstrap values; missing values indicate support below 70 (bootstrap). Each terminal includes the following information: GenBank code and species name. Highlighted branches indicated new sequences and excluded species of the *Pristimantis myersi* species group.

10.7717/peerj.14715/supp-3 Supplemental Information 3 Maximum likelihood tree (742 terminals) of *Pristimantis* + *Pristimantis myersi* group + outgroup of DNA sequences of the mitochondrial gene 16S Node support is expressed in Bootstrap values; missing values indicate support below 70 (bootstrap). Each terminal includes the following information: GenBank code, species name and voucher number. Highlighted branches indicated sequences of *Pristimantis myersi* group + close relatives clade + excluded species.

10.7717/peerj.14715/supp-4 Supplemental Information 4 Genetic distances (mitochondrial 16S) of *Pristimantis myersi group* and *P. verecundus* clades and related congeners Values are presented as percent distances calculated from uncorrected *p* values.

10.7717/peerj.14715/supp-5 Supplemental Information 5 Aligned dataset (742 terminals) of 16S sequences belonging to *Pristimantis* species

10.7717/peerj.14715/supp-6 Supplemental Information 6 Aligned dataset (215 terminals) of species in the *Pristimantis myersi* group and closely related taxa

10.7717/peerj.14715/supp-7 Supplemental Information 7 Genbank sequences (ON468258 to ON468432) of *Pristimantis myersi* group generated in this study

## Additional Information and Declarations

### Field Study Permissions

The following information was supplied relating to field study approvals (i.e., approving body and any reference numbers): The Ministerio de Ambiente y Agua de Ecuador, provided permits for investigation, handling and collection of specimens: MAE-DNB-CM-2018-0105.

### DNA Deposition

The following information was supplied regarding the deposition of DNA sequences: All sequences are available at GenBank: ON468258–ON468432.

### Data Availability

The following information was supplied regarding data availability: Raw data, including *Pristimantis myersi* group genetic sequences, are available in the Supplemental Files.
