# Supplementary material for: Unveiling the evolutionary relationships and the high cryptic diversity in Andean rainfrogs (Craugastoridae: Pristimantis myersi group)
Source: PeerJ. 2023 Mar 1;11:e14715. doi: 10.7717/peerj.14715 (PMC9985417; doi:10.7717/peerj.14715)
Supplement: Supplemental Information 1 — Species names, group/clade, GenBank accession numbers, voucher numbers, coordinates and localities of Pristimantis myersi species group, P. verecundus clade, P. devillei, P. boulengeri and P. leptolophus species groups. [file peerj-11-14715-s001.docx]

| **Species** | **Group/**  **Clade** | **GenBank Code** | **Voucher number** | **Locality** | **Latitude** | **Longitude** | **Elevation (m)** | **References** |
| --- | --- | --- | --- | --- | --- | --- | --- | --- |
| *Pristimantis* sp. 1 | ─ | JN104680.1 | UVC 15942 | Colombia: Cauca | 2.6381 N | 76.915 W | ─ | Genbank |
| *Pristimantis* sp. 1 | ─ | JN371037.1 | UVC 15943 | Colombia: Cauca | 2.6381 N | 76.915 W | ─ | Genbank |
| *Pristimantis jubatus* | ─ | JN104665.1 | UVC 15847 | Colombia: Cauca | 2.6642 N | 76.9025 W | ─ | Genbank |
| *Pristimantis jubatus* | ─ | JN104663.1 | UVC 15877 | Colombia: Cauca | 2.6381 N | 76.915 W | ─ | Genbank |
| *Pristimantis* sp. 2 | *P. verecundus* | EF493686 | QCAZ 12410 | Ecuador: Cotopaxi: Reserva Otonga | -0,418899 | -79,0039 | 1500 | Genbank |
| *Pristimantis* sp. 3 | *P. verecundus* | ON468258 | *JMG 675* | Ecuador: Carchi: Vía Ibarra-San Lorenzo | 0.84365 N | 78.22812 W | 2316 | Our study |
| *Pristimantis* sp. 4 | *P. verecundus* | ON468259 | DHMECN 13988 | Ecuador: Carchi: El Pailón | 0.98915 N | 78.23087 W | 1385 | Our study |
| *Pristimantis* sp. 5 | *P. verecundus* | ON468260 | DHMECN 14985 | Ecuador: Carchi: Maldonado | 0.894 N | 78.1176 W | ─ | Our study |
| *Pristimantis* sp. 5 | *P. verecundus* | ON468261 | DHMECN 14858 | Ecuador: Carchi: Maldonado | 0.8609 N | 78.2724 W | ─ | Our study |
| *P. celator* | *P. verecundus* | EF493685.1 | KU 177684 | Ecuador: Carchi: Maldonado | 0,83 N | 78,02 W | ─ | Genbank |
| *P. mutabilis* | *P. verecundus* | KM675457.1 | MZUTI 2191 | Ecuador: Pichincha: Rerserva Las Gralarias | 0.00954° S | 78.7346° W | 2030 | Genbank |
| *P. mutabilis* | *P. verecundus* | KM675458.1 | MZUTI 2190 | Ecuador: Pichincha: Rerserva Las Gralarias | 00.00843° S | 78.7305° W | 2063 | Genbank |
| *P. mutabilis* | *P. verecundus* | KM675460.1 | MZUTI 912 | Ecuador: Imbabura: Reserva Los Cedros | 0.3197 N | 78.7858 W | 1880 | Genbank |
| *P. mutabilis* | *P. verecundus* | KM675462.1 | MZUTI 910 | Ecuador: Imbabura: Reserva Los Cedros | 0.3197 N | 78.7858 W | 1880 | Genbank |
| *P. mutabilis* | *P. verecundus* | KM675461.1 | MZUTI 911 | Ecuador: Imbabura: Reserva Los Cedros | 0.3197 N | 78.7858 W | 1880 | Genbank |
| *P. mutabilis* | *P. verecundus* | KM675463.1 | MZUTI 909 | Ecuador: Imbabura: Reserva Los Cedros | 0.3197 N | 78.7858 W | 1880 | Genbank |
| *P. mutabilis* | *P. verecundus* | KM675459.1 | MZUTI 913 | Ecuador: Imbabura: Reserva Los Cedros | 0.3197 N | 78.7858 W | 1880 | Genbank |
| *P. mutabilis* | *P. verecundus* | ON468262 | ZSFQ 0504 | Ecuador: Imbabura: Manduriacu | 0.3099 N | 78.8567 W | 1264 | Our study |
| *P. mutabilis* | *P. verecundus* | ON468263 | *2019 027* | Ecuador: Imbabura: Manduriacu | 0.3099 N | 78.8567 W | 12O2 | Our study |
| *P. verecundus* | *P. verecundus* | ON468264 | DHMECN 15007 | Ecuador: Carchi: Reserva Dracula | 0.891944 N | 78.20308 W | 2150 | Our study |
| *P. verecundus* | *P. verecundus* | ON468265 | DHMECN 15188 | Ecuador: Carchi: Reserva Dracula | 0.884869 N | 78.204607 W | 1952 | Our study |
| *P. verecundus* | *P. verecundus* | ON468266 | DHMECN 12599 | Ecuador: Carchi: Reserva Dracula | 0.898426 N | 78.207942 W | 1600 | Our study |
| *P. verecundus* | *P. verecundus* | ON468267 | DHMECN 15189 | Ecuador: Carchi: Reserva Dracula | 0.885865 N | 78.207556 W | 2062 | Our study |
| *Pristimantis* sp. 6 | *P. verecundus* | ON468268 | DHMECN 14006 | Ecuador: Carchi: El Pailón | 0.984504 N | 78.224939 W | 1495 | Our study |
| *Pristimantis* sp. 7 | *P. verecundus* | ON468269 | DHMECN 13984 | Ecuador: Carchi: El Pailón | 0.989154 N | 78.230878 W | 1385 | Our study |
| *Pristimantis* sp. 8 | *P. verecundus* | ON468270 | *JMG 253* | Ecuador: Esmeraldas: Canande | 0.5258 N | 79.2088 W | 310 | Our study |
| *Pristimantis* sp. 8 | *P. verecundus* | ON468271 | *JMG 255* | Ecuador: Esmeraldas: Canande | 0.5258 N | 79.2088 W | 310 | Our study |
| *Pristimantis* sp. 9 | *P. verecundus* | ON468272 | ZSFQ 0503 | Ecuador: Imbabura: Manduriacu | 0.3099 N | 78.8567 W | 12O2 | Our study |
| *Pristimantis* sp. 9 | *P. verecundus* | ON468273 | *219 011* | Ecuador: Imbabura: Manduriacu | 0.3099 N | 78.8567 W | 12O2 | Our study |
| *Pristimantis* sp. 9 | *P. verecundus* | ON468274 | ZSFQ 0506 | Ecuador: Imbabura: Manduriacu | 0.3099 N | 78.8567 W | 1563 | Our study |
| *Pristimantis* sp. 9 | *P. verecundus* | ON468275 | ZSFQ 0505 | Ecuador: Imbabura: Manduriacu | 0.3099 N | 78.8567 W | 1588 | Our study |
| *Pristimantis* sp. 9 | *P. verecundus* | ON468276 | ZSFQ 0485 | Ecuador: Imbabura: Manduriacu | 0.3099 N | 78.8567 W | 1515 | Our study |
| *Pristimantis* sp. 10 | *P. verecundus* | ON468277 | MZUTI 3903 | Ecuador: Pichincha: Mashpi Lodge | 0.16075 N | 78.85611 W | 1279 | Our study |
| *Pristimantis* sp. 10 | *P. verecundus* | ON468278 | MZUTI 3764 | Ecuador: Pichincha: Mashpi Lodge | 0.16075 N | 78.85611 W | 1279 | Our study |
| *Pristimantis* sp. 10 | *P. verecundus* | ON468279 | MZUTI 3922 | Ecuador: Pichincha: Mashpi Lodge | 0.16075 N | 78.85611 W | 1279 | Our study |
| *Pristimantis* sp. 10 | *P. verecundus* | KM675445.1 | MZUTI 2114 | Ecuador: Pichincha: Mindo | 0.0285 S | 78.766 W | 1521 | Genbank |
| *Pristimantis* sp. 10 | *P. verecundus* | KM675446.1 | MZUTI 541 | Ecuador: Pichincha: Mindo | 0.07919 S | 78.76336 W | 1404 | Genbank |
| *Pristimantis* sp. 10 | *P. verecundus* | KM675447.1 | MZUTI 540 | Ecuador: Pichincha: Mindo | 0.07919 S | 78.76336 W | 1404 | Genbank |
| *Pristimantis* sp. 10 | *P. verecundus* | KM675448.1 | MZUTI 539 | Ecuador: Pichincha: Mindo | 0.0285 S | 78.766 W | 1521 | Genbank |
| *Pristimantis* sp. 10 | *P. verecundus* | KM675465.1 | MZUTI 635 | Ecuador: Pichincha: Cordillera de Chontilla | 0.112 S | 77.398 W | 1241 | Genbank |
| *Pristimantis* sp. 10 | *P. verecundus* | KM675466.1 | MZUTI 634 | Ecuador: Pichincha: Cordillera de Chontilla | 0.112 S | 77.398 W | 1241 | Genbank |
| *Pristimantis* sp. 10 | *P. verecundus* | KM675464.1 | MZUTI 636 | Ecuador: Pichincha: Cordillera de Chontilla | 0.112 S | 77.398 W | 1241 | Genbank |
| *Pristimantis* sp. 10 | *P. verecundus* | KM675467.1 | MZUTI 633 | Ecuador: Pichincha: Cordillera de Chontilla | 0.112 S | 77.398 W | 1241 | Genbank |
| *P. pyrrhomerus* | *P. myersi* | ON468283 | MZUTI 1943 | Ecuador: Cotopaxi: ± 2 km E of Pilaló | 0.9464166 S | 78.973305 W | 2720 | Our study |
| *P. pyrrhomerus* | *P. myersi* | ON468284 | MZUTI 1942 | Ecuador: Cotopaxi: ± 2 km E of Pilaló | 0.9464166 S | 78.973305 W | 2720 | Our study |
| *P. pyrrhomerus* | *P. myersi* | ON468285 | MZUTI 1941 | Ecuador: Cotopaxi: ± 2 km E of Pilaló | 0.9464166 S | 78.973305 W | 2720 | Our study |
| *P. sirnigeli* | *P. myersi* | ON468280 | MZUTI 1825 | Ecuador: Pichincha: Verdecocha | 0.10444 S | 78.610055 W | 3084 | Our study |
| *P. sirnigeli* | *P. myersi* | ON468281 | MZUTI 1827 | Ecuador: Pichincha: Verdecocha | 0.10444 S | 78.610055 W | 3084 | Our study |
| *P. sirnigeli* | *P. myersi* | ON468282 | MZUTI 1826 | Ecuador: Pichincha: Verdecocha | 0.103333 S | 78.610277 W | 3078 | Our study |
| *Pristimantis* sp. 11 | *P. myersi* | ON468286 | MZUTI 1928 | Ecuador: Cotopaxi: Sigchos | 0.6836944 S | 78.900305 W | 2803 | Our study |
| *Pristimantis* sp. 11 | *P. myersi* | ON468287 | MZUTI 1927 | Ecuador: Cotopaxi: Sigchos | 0.6836944 S | 78.900305 W | 2803 | Our study |
| *Pristimantis* sp. 11 | *P. myersi* | ON468288 | MZUTI 1926 | Ecuador: Cotopaxi: Sigchos | 0.6836944 S | 78.900305 W | 2803 | Our study |
| *Pristimantis* sp. 11 | *P. myersi* | ON468289 | MZUTI 1925 | Ecuador: Cotopaxi: Sigchos | 0.6836944 S | 78.900305 W | 2803 | Our study |
| *Pristimantis* sp. 12 | *P. myersi* | ON468290 | DHMECN 13354 | Ecuador: Carchi: Virgen Negra | 0.6699857 N | 77.590419 W | ─ | Our study |
| *Pristimantis* sp. 12 | *P. myersi* | ON468291 | DHMECN 13347 | Ecuador: Carchi: Virgen Negra | 0.6699857 N | 77.590419 W | ─ | Our study |
| *Pristimantis* sp. 12 | *P. myersi* | ON468292 | DHMECN 13649 | Ecuador: Carchi: Loma la Esperanza | 0.531531 N | 77.762939 W | ─ | Our study |
| *Pristimantis* sp. 12 | *P. myersi* | ON468293 | DHMECN 13648 | Ecuador: Carchi: Loma la Esperanza | 0.531531 N | 77.762939 W | ─ | Our study |
| *Pristimantis* sp. 12 | *P. myersi* | ON468294 | DHMECN 13339 | Ecuador: Carchi: Morán | 0.769279 N | 78.053656 W | ─ | Our study |
| *Pristimantis* sp. 12 | *P. myersi* | ON468295 | ZSFQ 4448 | Ecuador: Carchi: Camino tufiño maldonado | 0.8028 N | 78.0047 W | 3362 | Our study |
| *Pristimantis* sp. 12 | *P. myersi* | ON468296 | ZSFQ 4453 | Ecuador: Carchi: Camino tufiño maldonado | 0.8028 N | 78.0047 W | 3362 | Our study |
| *Pristimantis* sp. 12 | *P. myersi* | ON468297 | ZSFQ 4450 | Ecuador: Carchi: Camino tufiño maldonado | 0.8028 N | 78.0047 W | 3362 | Our study |
| *Pristimantis* sp. 12 | *P. myersi* | ON468298 | ZSFQ 4447 | Ecuador: Carchi: Camino tufiño maldonado | 0.8028 N | 78.0047 W | 3362 | Our study |
| *Pristimantis* sp. 12 | *P. myersi* | ON468299 | ZSFQ 4541 | Ecuador: Carchi: Morán | 0.769279 N | 78.053656 W | 3056 | Our study |
| *Pristimantis* sp. 13 | *P. myersi* | ON468300 | QCAZ 13771 | Ecuado: Bolívar: Bosque Protector Cashca Totoras | 1.718 S | 78.9766 W | 2900 | Our study |
| *Pristimantis* sp. 13 | *P. myersi* | ON468301 | QCAZ 13769 | Ecuado: Bolívar: Bosque Protector Cashca Totoras | 1.718 S | 78.9766 W | 2900 | Our study |
| *Pristimantis* sp. 13 | *P. myersi* | EF493683.1 | KU 218030 | Ecuador: Bolívar: Bosque Protector Cashca Totoras | 1,7 S | 78,88 W | ─ | Genbank |
| *P. leoni* | *P. myersi* | ON468302 | QCAZ 42125 | Ecuador: Imbabura: Nudo norte de Mojanda | 0.1663 S | 78.2913 W | 3385 | Our study |
| *P. leoni* | *P. myersi* | ON468303 | MZUTI 1811 | Ecuador: Imbabura: Laguna de Mojanda | 0.1605 S | 78.282694 W | 3557 | Our study |
| *P. leoni* | *P. myersi* | ON468304 | MZUTI 1809 | Ecuador: Imbabura: Laguna de Mojanda | 0.1605 S | 78.282694 W | 3557 | Our study |
| *P. leoni* | *P. myersi* | ON468305 | DHMECN 7287 | Ecuador: Pichincha: Yanacocha | 0.103897 S | 78.584998 W | 3000 | Our study |
| *P. leoni* | *P. myersi* | ON468306 | MZUTI 1799 | Ecuador: Pichincha: Verdecocha | 0.104444 S | 78.610055 W | 3084 | Our study |
| *P. leoni* | *P. myersi* | ON468307 | DHMECN 7285 | Ecuador: Pichincha: Yanacocha | 0.1039 S | 78.585 W | 3000 | Our study |
| *P. leoni* | *P. myersi* | ON468308 | MZUTI 1818 | Ecuador: Pichincha: Verdecocha | 0.10333 S | 78.61028 W | 3078 | Our study |
| *P. leoni* | *P. myersi* | ON468309 | DHMECN 8762 | Ecuador: Pichincha: Valle del Toaza | 0.2011 S | 78.6231 W | 3420 | Our study |
| *P. leoni* | *P. myersi* | ON468310 | MZUTI 1819 | Ecuador: Pichincha: Verdecocha | 0.103333 S | 78.610277 W | 3078 | Our study |
| *P. leoni* | *P. myersi* | ON468311 | DHMECN 8756 | Ecuador: Pichincha: Valle del Toaza | 0.2011 S | 78.6231 W | 3420 | Our study |
| *P. leoni* | *P. myersi* | ON468312 | DHMECN 8759 | Ecuador: Pichincha: Valle del Toaza | 0.2011 S | 78.6231 W | 3420 | Our study |
| *P. leoni* | *P. myersi* | ON468313 | DHMECN 8757 | Ecuador: Pichincha: Valle del Toaza | 0.2011 S | 78.6231 W | 3420 | Our study |
| *P. leoni* | *P. myersi* | ON468314 | DHMECN 8760 | Ecuador: Pichincha: Valle del Toaza | 0.2011 S | 78.6231 W | 3420 | Our study |
| *P. munozi* | *P. myersi* | ON468315 | DHMECN 4938 | Ecuador: Pichincha: La Victoria | 0.209 S | 78.7229 W | 2048 | Our study |
| *P. munozi* | *P. myersi* | ON468316 | MZUTI 1783 | Ecuador: Pichincha: Verdecocha | 0.0963611 S | 78.60425 W | 2855 | Our study |
| *P. munozi* | *P. myersi* | ON468317 | MZUTI 1782 | Ecuador: Pichincha: Verdecocha | 0.0963611 S | 78.60425 W | 2855 | Our study |
| *P. munozi* | *P. myersi* | ON468318 | MZUTI 1784 | Ecuador: Pichincha: Verdecocha | 0.0963611 S | 78.60425 W | 2855 | Our study |
| *P. munozi* | *P. myersi* | ON468319 | MZUTI 1779 | Ecuador: Pichincha: Verdecocha | 0.0963611 S | 78.60425 W | 2855 | Our study |
| *Pristimantis* sp. 14 | *P. myersi* | ON468320 | DHMECN 13329 | Ecuador: Carchi: Cerro la Bretaña | 0.568867 N | 77.71422 W | ─ | Our study |
| *Pristimantis* sp. 14 | *P. myersi* | ON468321 | DHMECN 13356 | Ecuador: Carchi: Cordillera de la Virgen Negra | 0.669985747 N | 77.5904199 W | ─ | Our study |
| *Pristimantis* sp. 14 | *P. myersi* | ON468322 | DHMECN 13355 | Ecuador: Carchi: Cordillera de la Virgen Negra | 0.669985747 N | 77.5904199 W | ─ | Our study |
| *Pristimantis* sp. 14 | *P. myersi* | ON468323 | DHMECN 13645 | Ecuador: Carchi: Loma La Esperanza | 0.531531 N | 77.762939 W | ─ | Our study |
| *Pristimantis* sp. 14 | *P. myersi* | ON468324 | DHMECN 13327 | Ecuador: Carchi: Cerro la Bretaña | 0.568867 N | 77.71422 W | ─ | Our study |
| *Pristimantis* sp. 14 | *P. myersi* | ON468325 | DHMECN 13644 | Ecuador: Carchi: Loma La Esperanza | 0.531531 N | 77.762939 W | ─ | Our study |
| *Pristimantis* sp. 14 | *P. myersi* | ON468326 | DHMECN 13642 | Ecuador: Carchi: Loma La Esperanza | 0.531531 N | 77.762939 W | ─ | Our study |
| *Pristimantis* sp. 14 | *P. myersi* | ON468327 | ZSFQ 4511 | Ecuador: Carchi: Loma La Esperanza | 0.531531 N | 77.762939 W | 3019 | Our study |
| *Pristimantis* sp. 14 | *P. myersi* | ON468328 | ZSFQ 4513 | Ecuador: Carchi: Loma La Esperanza | 0.531531 N | 77.762939 W | 3118 | Our study |
| *Pristimantis* sp. 15 | *P. myersi* | ON468329 | ZSFQ 4431 | Ecuador: Carchi: San Francisco | 0.65 N | 77.783333 W | 3411 | Our study |
| *Pristimantis* sp. 15 | *P. myersi* | ON468330 | DHMECN 13633 | Ecuador: Carchi: San Francisco | 0.65 N | 77.783333 W | ─ | Our study |
| *Pristimantis* sp. 15 | *P. myersi* | ON468331 | DHMECN 13635 | Ecuador: Carchi: San Francisco | 0.65 N | 77.783333 W | ─ | Our study |
| *Pristimantis* sp. 15 | *P. myersi* | ON468332 | DHMECN 13634 | Ecuador: Carchi: San Francisco | 0.65 N | 77.783333 W | ─ | Our study |
| *P. ocreatus* | *P. myersi* | ON468333 | ZSFQ 4546 | Ecuador: Carchi: Morán | 0.769279 N | 78.053656 W | 3848 | Our study |
| *P. ocreatus* | *P. myersi* | ON468334 | QCAZ 43161 | Ecuador: Carchi: Vía Tulcan Maldonado | 0.791972 N | 77.90691 W | 3817 | Our study |
| *P. ocreatus* | *P. myersi* | ON468335 | DHMECN 13650 | Ecuador: Carchi: Complejo Aguas Ediondas | 0.810558 N | 77.904306 W | 3595 | Our study |
| *P. ocreatus* | *P. myersi* | ON468336 | QCAZ 42111 | Ecuador: Imbabura: Laguna de Mojanda | 0.12667 N | 78.25606 W | 3834 | Our study |
| *P. ocreatus* | *P. myersi* | ON468337 | DHMECN 13669 | Ecuador: Carchi: Complejo Aguas Ediondas | 0.810558 N | 77.904306 W | 3595 | Our study |
| *P. ocreatus* | *P. myersi* | ON468338 | QCAZ 43162 | Ecuador: Carchi: Vía Tulcan Maldonado | 0.793861 N | 77.91111 W | 3841 | Our study |
| *P. ocreatus* | *P. myersi* | ON468339 | ZSFQ 4456 | Ecuador: Carchi: Potrerillos | 0.804305 N | 77.000861 W | 3785 | Our study |
| *P. ocreatus* | *P. myersi* | ON468340 | ZSFQ 4454 | Ecuador: Carchi: Potrerillos | 0.804305 N | 77.000861 W | 3785 | Our study |
| *P. ocreatus* | *P. myersi* | ON468341 | ZSFQ 4457 | Ecuador: Carchi: Potrerillos | 0.804305 N | 77.000861 W | 3785 | Our study |
| *P. ocreatus* | *P. myersi* | ON468342 | ZSFQ 4455 | Ecuador: Carchi: Potrerillos | 0.804305 N | 77.000861 W | 3785 | Our study |
| *P. ocreatus* | *P. myersi* | JX564889.1 |  | Ecuador | ─ | ─ | ─ | Genbank |
| *P. ocreatus* | *P. myersi* | EF493682.1 | KU 208508 | Ecuador: Carchi: 26.6 km W Tulcán | 0,7892649 | -77,89259 | ─ | Genbank |
| *P. gladiator* | *P. myersi* | ON468343 | MZUTI 1131 | Ecuador: Napo: Papallacta | 0.37639 S | 78.07471 W | 2708 | Our study |
| *P. gladiator* | *P. myersi* | ON468344 | MZUTI 1117 | Ecuador: Napo: Papallacta | 0.37575 S | 78.12173 W | 2973 | Our study |
| *P. gladiator* | *P. myersi* | ON468345 | QCAZ 18875 | Ecuador: Napo: Guango Lodge | 0.378055 S | 78.07416 W | 2717 | Our study |
| *P. gladiator* | *P. myersi* | ON468346 | MZUTI 1123 | Ecuador: Napo: Papallacta | 0.36773 S | 78.09313 W | 2820 | Our study |
| *P. gladiator* | *P. myersi* | ON468347 | QCAZ 18874 | Ecuador: Napo: Guango Lodge | 0.378055 S | 78.07416 W | 2717 | Our study |
| *P. gladiator* | *P. myersi* | ON468348 | MZUTI 1122 | Ecuador: Napo: Papallacta | 0.36773 S | 78.09313 W | 2820 | Our study |
| *P. gladiator* | *P. myersi* | ON468349 | MZUTI 1214 | Ecuador: Napo: Papallacta | 0.36773 S | 78.09313 W | 2820 | Our study |
| *P. gladiator* | *P. myersi* | ON468350 | MZUTI 1118 | Ecuador: Napo: Papallacta | 0.37575 S | 78.12173 W | 2973 | Our study |
| *Pristimantis* sp. 16 | *P. myersi* | ON468351 | DHMECN 15824 | Ecuador: Tungurahua: Reserva EcoMinga | 1.451638889 S | 78.30822222 W | ─ | Our study |
| *Pristimantis* sp. 16 | *P. myersi* | ON468352 | DHMECN 14702 | Ecuador: Tungurahua: Reserva EcoMinga | 1.420639 S | 78.387521 W | 3122 | Our study |
| *Pristimantis* sp. 16 | *P. myersi* | ON468353 | DHMECN 14701 | Ecuador: Tungurahua: Reserva EcoMinga | 1.420639 S | 78.387521 W | 3122 | Our study |
| *Pristimantis* sp. 17 | *P. myersi* | ON468354 | DHMECN 14450 | Ecuador: Tungurahua: Reserva EcoMinga | 1.3701 S | 78.2683 W | 2781 | Our study |
| *Pristimantis* sp. 17 | *P. myersi* | ON468355 | DHMECN 14446 | Ecuador: Tungurahua: Reserva EcoMinga | 1.432352846 S | 78.27286411 W | 2220 | Our study |
| *Pristimantis* sp. 17 | *P. myersi* | ON468356 | QCAZ 40808 | Ecuador: Napo: Cordillera Guacamayos | 0.624028 S | 77.84086 W | 2294 | Our study |
| *Pristimantis* sp. 17 | *P. myersi* | ON468357 | QCAZ 41305 | Ecuador: Napo: Parque Nacional Sumaco | 0.56964 S | 77.59412 W | 2300 | Our study |
| *Pristimantis* sp. 17 | *P. myersi* | ON468358 | QCAZ 41260 | Ecuador: Napo: Camino al volcan Sumaco | 0.56396 S | 77.61548 W | 2775 | Our study |
| *P. festae* | *P. myersi* | ON468359 | QCAZ 11677 | Ecuador: Imbabura: Laguna de Puruhanta | 0.2050097 N | 77.95454 W | 2800 | Our study |
| *P. festae* | *P. myersi* | ON468360 | QCAZ 52629 | Ecuador: Imbabura: Hacienda Zuleta | 0.19441 N | 78.06548 W | 2955 | Our study |
| *P. festae* | *P. myersi* | ON468361 | QCAZ 52628 | Ecuador: Imbabura: Hacienda Zuleta | 0.19441 N | 78.06548 W | 2955 | Our study |
| *P. festae* | *P. myersi* | ON468362 | QCAZ 49755 | Ecuador: Pichincha: Reserva Ecológica Cayambe-Coca | 0.10571 N | 77.96307 W | 3140 | Our study |
| *P. festae* | *P. myersi* | ON468363 | ZSFQ 4519 | Ecuador: Carchi: Loma La Esperanza | 0.531531 N | 77.762939 W | 3118 | Our study |
| *P. festae* | *P. myersi* | ON468364 | ZSFQ 4425 | Ecuador: Carchi: San Francisco | 0.65 N | 77.783333 W | 3591 | Our study |
| *P. festae* | *P. myersi* | ON468365 | DHMECN 13641 | Ecuador: Carchi: San Francisco | 0.65 N | 77.783333 W | ─ | Our study |
| *P. festae* | *P. myersi* | ON468366 | DHMECN 13640 | Ecuador: Carchi: San Francisco | 0.65 N | 77.783333 W | ─ | Our study |
| *P. festae* | *P. myersi* | ON468367 | MZUTI 4813 | Ecuador: Pichincha: Cayambe | 0.004167 N | 78.025278 W | 4240 | Our study |
| *P. festae* | *P. myersi* | ON468368 | QCAZ 17950 | Ecuador: Napo: Papallacta | 0.370329 S | 78.19231 W | 3730 | Our study |
| *P. festae* | *P. myersi* | ON468369 | QCAZ 49680 | Ecuador: Napo: Paramo de Guamani | 0.34627 S | 78.19969 W | 3950 | Our study |
| *P. festae* | *P. myersi* | ON468370 | QCAZ 16405 | Ecuador: Napo: Paramo de Guamani | 0.359018 S | 78.19029 W | 4000 | Our study |
| *P. festae* | *P. myersi* | ON468371 | QCAZ 16404 | Ecuador: Napo: Paramo de Guamani | 0.359018 S | 78.19029 W | 4000 | Our study |
| *P. festae* | *P. myersi* | ON468372 | QCAZ 13667 | Ecuador: Imbabura: Laguna de Mojanda, 3 km E | 0.129727 N | 78.23995 W | 3500 | Our study |
| *P. festae* | *P. myersi* | ON468373 | QCAZ 13677 | Ecuador: Imbabura: Laguna de Mojanda, 3 km E | 0.129727 N | 78.23995 W | 3500 | Our study |
| *P. festae* | *P. myersi* | ON468374 | MZUTI 1807 | Ecuador: Imbabura: Laguna de Mojanda | 0.1605 S | 78.28269 W | 3557 | Our study |
| *P. festae* | *P. myersi* | ON468375 | QCAZ 43164 | Ecuador: Carchi: Vía Tulcan Maldonado | 0.793861 N | 77.91111 W | 3841 | Our study |
| *P. festae* | *P. myersi* | ON468376 | QCAZ 42116 | Ecuador: Imbabura: Laguna de Mojanda | 0.12667 N | 78.25606 W | 3834 | Our study |
| *P. festae* | *P. myersi* | ON468377 | QCAZ 42109 | Ecuador: Imbabura: Laguna de Mojanda | 0.12667 N | 78.25606 W | 3834 | Our study |
| *P. gralarias* | *P. myersi* | MH306193.1 | MZUTI 1466 | Ecuador: Pichincha: Reserva Las Gralarias | 0.0275 S | 78.7048 W | 2192 | Genbank |
| *Pristimantis* sp. 18 | *P. myersi* | MH516183.1 | QCAZ 25589 | Ecuador: Orellana: Dayuma | ─ | ─ | ─ | Genbank |
| *Pristimantis* sp. 18 | *P. myersi* | EF493684.1 | KU 218227 | Ecuador: Carchi: 51.3 km W Tulcán | 0,91 | -78,09 | ─ | Genbank |
| *Pristimantis* sp. 18 | *P. myersi* | ON468378 | *TH 668* | Ecuador: Carchi: La Centella | 0.81436 N | 78.01497 W | 2806 | Our study |
| *Pristimantis* sp. 18 | *P. myersi* | ON468379 | *TH 639* | Ecuador: Carchi: La Centella | 0.81436 N | 78.01497 W | 2806 | Our study |
| *Pristimantis* sp. 18 | *P. myersi* | ON468380 | DHMECN 13336 | Ecuador: Carchi: Morán | 0.769279 N | 78.0536569 W | ─ | Our study |
| *Pristimantis* sp. 18 | *P. myersi* | ON468381 | DHMECN 13332 | Ecuador: Carchi: Morán | 0.769279 N | 78.0536569 W | ─ | Our study |
| *Pristimantis* sp. 18 | *P. myersi* | ON468382 | DHMECN 13328 | Ecuador: Carchi: Cerro la Bretaña | 0.568867 N | 77.71422 W | ─ | Our study |
| *Pristimantis hectus* | *P. myersi* | ON468383 | DHMECN 14861 | Ecuador: Carchi: Río Chinambi | 0.860988 N | 78.269545 W | 2177 | Our study |
| *P. hectus* | *P. myersi* | ON468384 | DHMECN 14888 | Ecuador: Carchi: Río Chinambi | 0.860638 N | 78.273316 W | 2172 | Our study |
| *P. hectus* | *P. myersi* | ON468385 | DHMECN 14847 | Ecuador: Carchi: Río Chinambi | 0.862668 N | 78.267578 W | 2184 | Our study |
| *P. hectus* | *P. myersi* | ON468386 | DHMECN 14887 | Ecuador: Carchi: Río Chinambi | 0.860973 N | 78.274114 W | 2176 | Our study |
| *P. hectus* | *P. myersi* | ON468387 | DHMECN 15167 | Ecuador: Carchi: Reserva Dracula | 0.885865 N | 78.207556 W | 1976 | Our study |
| *P. hectus* | *P. myersi* | ON468388 | DHMECN 15169 | Ecuador: Carchi: Reserva Dracula | 0.885043 N | 78.207548 W | 2000 | Our study |
| *P. hectus* | *P. myersi* | ON468389 | DHMECN 15172 | Ecuador: Carchi: Reserva Dracula | 0.882831 N | 78.209597 W | 2107 | Our study |
| *P. hectus* | *P. myersi* | ON468390 | DHMECN 14993 | Ecuador: Carchi: Reserva Dracula | 0.891944 N | 78.20308 W | 2000 | Our study |
| *P. hectus* | *P. myersi* | ON468391 | DHMECN 14991 | Ecuador: Carchi: Reserva Dracula | 0.891944 N | 78.20308 W | 2000 | Our study |
| *P. hectus* | *P. myersi* | ON468392 | DHMECN 15165 | Ecuador: Carchi: Reserva Dracula | 0.886697 N | 78.207296 W | 1898 | Our study |
| *P. hectus* | *P. myersi* | ON468393 | DHMECN 14994 | Ecuador: Carchi: Reserva Dracula | 0.891944 N | 78.20308 W | 2140 | Our study |
| *P. onorei* | *P. myersi* | ON468394 | QCAZ 12288 | Ecuador: Cotopaxi: Bosque Integral Otonga | 0.419443 S | 79.00333 W | 2000 | Our study |
| *P. lucidosignatus* | *P. myersi* | ON468395 | MZUTI 2092 | Ecuador: Cotopaxi: Bosque Integral Otonga | 0.4155 S | 79.0048 W | 2115 | Our study |
| *P. lucidosignatus* | *P. myersi* | ON468396 | MZUTI 2063 | Ecuador: Cotopaxi: Bosque Integral Otonga | 0.4155 S | 79.0048 W | 2115 | Our study |
| *P. lucidosignatus* | *P. myersi* | ON468397 | MZUTI 2094 | Ecuador: Cotopaxi: Bosque Integral Otonga | 0.4155 S | 79.0048 W | 2115 | Our study |
| *P. lucidosignatus* | *P. myersi* | ON468398 | MZUTI 2095 | Ecuador: Cotopaxi: Bosque Integral Otonga | 0.4155 S | 79.0048 W | 2115 | Our study |
| *P. lucidosignatus* | *P. myersi* | ON468399 | MZUTI 2070 | Ecuador: Cotopaxi: Bosque Integral Otonga | 0.4155 S | 79.0048 W | 2115 | Our study |
| *P. lucidosignatus* | *P. myersi* | ON468400 | MZUTI 2088 | Ecuador: Cotopaxi: Bosque Integral Otonga | 0.4155 S | 79.0048 W | 2115 | Our study |
| *P. lucidosignatus* | *P. myersi* | ON468401 | MZUTI 2093 | Ecuador: Cotopaxi: Bosque Integral Otonga | 0.4155 S | 79.0048 W | 2115 | Our study |
| *P. lucidosignatus* | *P. myersi* | ON468402 | MZUTI 2075 | Ecuador: Cotopaxi: Bosque Integral Otonga | 0.4155 S | 79.0048 W | 2115 | Our study |
| *P. lucidosignatus* | *P. myersi* | ON468403 | MZUTI 2066 | Ecuador: Cotopaxi: Bosque Integral Otonga | 0.4155 S | 79.0048 W | 2115 | Our study |
| *P. onorei* | *P. myersi* | ON468404 | MZUTI 4241 | Ecuador: Imbabura: Toisan | 0.5032 N | 78.57 N | 2575 | Our study |
| *P. onorei* | *P. myersi* | ON468405 | MZUTI 4244 | Ecuador: Imbabura: Toisan | 0.5047 N | 78.55 N | 2558 | Our study |
| *P. onorei* | *P. myersi* | ON468406 | MZUTI 4243 | Ecuador: Imbabura: Toisan | 0.5044 N | 78.5461 N | 2575 | Our study |
| *P. onorei* | *P. myersi* | ON468407 | MZUTI 1467 | Ecuador: Pichincha: Reserva Las Gralarias | 0.02557 S | 78.70391 W | 2136 | Our study |
| *P. onorei* | *P. myersi* | ON468408 | MZUTI 2035 | Ecuador: Pichincha: Reserva Las Gralarias | 0.02557 S | 78.70391 W | 2136 | Our study |
| *P. onorei* | *P. myersi* | ON468409 | MZUTI 2031 | Ecuador: Pichincha: Reserva Las Gralarias | 0.02557 S | 78.70391 W | 2136 | Our study |
| *P. onorei* | *P. myersi* | ON468410 | MZUTI 2032 | Ecuador: Pichincha: Reserva Las Gralarias | 0.02557 S | 78.70391 W | 2136 | Our study |
| *P. onorei* | *P. myersi* | ON468411 | MZUTI 2024 | Ecuador: Pichincha: Reserva Las Gralarias | 0.02557 S | 78.70391 W | 2136 | Our study |
| *P. onorei* | *P. myersi* | ON468412 | MZUTI 2038 | Ecuador: Pichincha: Reserva Las Gralarias | 0.02557 S | 78.70391 W | 2136 | Our study |
| *P. onorei* | *P. myersi* | ON468413 | MZUTI 2037 | Ecuador: Pichincha: Reserva Las Gralarias | 0.02557 S | 78.70391 W | 2136 | Our study |
| *P. onorei* | *P. myersi* | ON468414 | MZUTI 1465 | Ecuador: Pichincha: Reserva Las Gralarias | 0.02557 S | 78.70391 W | 2136 | Our study |
| *P. onorei* | *P. myersi* | ON468415 | MZUTI 1464 | Ecuador: Pichincha: Reserva Las Gralarias | 0.02557 S | 78.70391 W | 2136 | Our study |
| *P. onorei* | *P. myersi* | ON468416 | MZUTI 2030 | Ecuador: Pichincha: Reserva Las Gralarias | 0.02557 S | 78.70391 W | 2136 | Our study |
| *P. onorei* | *P. myersi* | ON468417 | MZUTI 2023 | Ecuador: Pichincha: Reserva Las Gralarias | 0.02557 S | 78.70391 W | 2136 | Our study |
| *P. onorei* | *P. myersi* | ON468418 | DHMECN 6813 | Ecuador: Pichincha: Bellavista | 0.024092 S | 78.708213 W | 2375 | Our study |
| *P. onorei* | *P. myersi* | ON468419 | DHMECN 6815 | Ecuador: Pichincha: Bellavista | 0.024092 S | 78.708213 W | 2375 | Our study |
| *P. onorei* | *P. myersi* | ON468420 | DHMECN 6821 | Ecuador: Pichincha: Bellavista | 0.024092 S | 78.708213 W | 2375 | Our study |
| *P. onorei* | *P. myersi* | ON468421 | DHMECN 6812 | Ecuador: Pichincha: Bellavista | 0.024092 S | 78.708213 W | 2375 | Our study |
| *Pristimantis floridus* | *P. myersi* | ─ | DHMECN8573 | Ecuador: Pichincha: Estación Chiquilpe | 0.02926 N | 78,604494 W | 3316 m | Frost, 2021; Lynch & Duellman, 1997 |
| *Pristimantis floridus* | *P. myersi* | ─ | USNM239683 | Ecuador: Pichincha: Río Blanco | 0.28999 N | 78,720000 W | ─ | Frost, 2021; Lynch & Duellman, 1997 |
| *Pristimantis floridus* | *P. myersi* | ─ | USNM239684 | Ecuador: Santo Domingo: San Miguel de Congomá | 0.310000 N | 79,250000 W | ─ | Frost, 2021; Lynch & Duellman, 1997 |
| *Pristimantis floridus* | *P. myersi* | ─ | DHMECN7234 | Ecuador: Pichincha: San Jose de Minas | 0.183333 N | 78,516662 W | 1800 m | Frost, 2021; Lynch & Duellman, 1997 |
| *P. albujai* | ─ | ON468422 | DHMECN 12246 | Ecuador: Morona Santiago: Río Blanco | 2.0793 S | 78.1604 W | 3650 | Our study |
| *P.sambalan* | ─ | ON468423 | DHMECN 12249 | Ecuador: Morona Santiago: Río Blanco | 2.2061 S | 78.4527 W | 3337 | Our study |
| *P. bicantus* | ─ | ON468424 | QCAZ 31988 | Ecuador: Napo: Cosanga | 0.5992 S | 77.8897 W | 2000 | Our study |
| *P. bicantus* | ─ | ON468425 | QCAZ 24653 | Ecuador: Morona Santiago: Chinguinda | 3.2004 S | 78.7631 W | 2521 | Our study |
| *P. bicantus* | ─ | ON468426 | QCAZ 37182 | Ecuador: Morona Santiago: Quebrada Cugusha | 2.2216 S | 78.2883 W | 1729 | Our study |
| *Pristimantis cf. bicantus* | ─ | ON468427 | QCAZ 45803 | Ecuador: Pastaza: Río Challuwa Yacu | 1.2762 S | 78.0725 W | 2266 | Our study |
| *Pristimantis cf. bicantus* | ─ | ON468428 | QCAZ 45805 | Ecuador: Pastaza: Río Challuwa Yacu | 1.2762 S | 78.0725 W | 2266 | Our study |
| *P. bicantus* | ─ | ON468429 | QCAZ 49035 | Ecuador: Morona Santiago: Bosque Protector Abanico | 2.2538 S | 78.1989 W | 1647 | Our study |
| *P. bicantus* | ─ | ON468430 | QCAZ 51561 | Ecuador: Cañar: Camunidad San Antonio de Juva | 2.4122 S | 78.696 W | 2451 | Our study |
| *P. nelsongalloi* | ─ | ON468431 | QCAZ 52464 | Ecuador: Tungurahua: Reserva Río Zuñac | 1.3674 S | 78.1457 W | 2140 | Our study |
| *P. nelsongalloi* | ─ | ON468432 | QCAZ 52466 | Ecuador: Tungurahua: Reserva Río Zuñac | 1.3674 S | 78.1457 W | 2140 | Our study |
| *P. quinquagesimus* | *P. devillei* | EF493690.1 | KU 179374 | Ecuador: Quebrada Zapadores | ─ | ─ | ─ | Genbank |
| *P. sobetes* | *P. devillei* | KM675454.1 | MZUTI 442 | Ecuador: Pichincha: Reserva Las Gralarias | ─ | ─ | ─ | Genbank |
| *P. thymalopsoides* | *P. devillei* | EF493514.1 | KU 177861 | Ecuador: Cotopaxi: Pilaló | ─ | ─ | ─ | Genbank |
| *P. devillei* | *P. devillei* | EF493688.1 | KU 217991 | Ecuador: Napo: Papallacta | ─ | ─ | ─ | Genbank |
| *P. pichincha* | *P. devillei* | EF493688.1 | QCAZ 11673 | Ecuador: Imbabura: Mojanda | ─ | ─ | ─ | Genbank |
| *P. vertebralis* | *P. devillei* | EF493689.1 | KU177972 | Ecuador: Imbabura: La Delicia | ─ | ─ | ─ | Genbank |
| *P. buckleyi* | *P. devillei* | EF493350.1 | KU 217836 | Ecuador: Carchi: Páramo El Ángel | ─ | ─ | ─ | Genbank |
| *P. curtipes* | *P. devillei* | EF493513.1 | KU 217871 | Ecuador: Cotopaxi: Bosque Pasochoa | ─ | ─ | ─ | Genbank |
| *P. yumbo* | *P. devillei* | MK881494.1 | QCAZ 52241 | Ecuador: Bosque Tandacato | ─ | ─ | ─ | Genbank |
| *P. truebae* | *P. devillei* | EF493512.1 | KU 218013 | Ecuador: Cotopaxi: Pilaló | ─ | ─ | ─ | Genbank |
| *P. gentryi* | *P. devillei* | EF493511.1 | KU 218109 | Ecuador: Cotopaxi: Pilaló | ─ | ─ | ─ | Genbank |
| *P. quantus* | *P. boulengeri* | JN104684.1 | UVC 15905 | Colombia: Cauca | ─ | ─ | ─ | Genbank |
| *P. brevifros* | *P. boulengeri* | JN370962.1 | UVC 15834 | Colombia: Cauca | ─ | ─ | ─ | Genbank |
| *P. angustilineatus* | *P. boulengeri* | JN104677.1 | UVC 15888 | Colombia: Cauca | ─ | ─ | ─ | Genbank |
| *P. dorsopictus* | *P. boulengeri* | KU724440.1 | MHUAA 8960 | Colombia: Antioquia | ─ | ─ | ─ | Genbank |
| *P. myops* | *P. boulengeri* | JN104682.1 | UVC 15824 | Colombia: Cauca | ─ | ─ | ─ | Genbank |
| *P. acatellelus* | *P. leptolophus* | JN104675.1 | UVC 15914 | Colombia: Meta | ─ | ─ | ─ | Genbank |
| *P. leptolophus* | *P. leptolophus* | KY494226.1 | JJS 093 | Colombia: Cauca | ─ | ─ | ─ | Genbank |
| *P. maculosus* | *P. leptolophus* | KY494240.1 | ICN 55760 | Colombia: Caldas | ─ | ─ | ─ | Genbank |
| *P. parectatus* | *P. leptolophus* | KY627807.1 | MHUAA 9946 | Colombia | ─ | ─ | ─ | Genbank |
| *P. scoloblepharus* | *P. leptolophus* | KY627801.1 | MHUAA 9873 | Colombia | ─ | ─ | ─ | Genbank |
| *P. leopardus* | *P. leptolophus* | KY627789.1 | MHUAA 7630 | Colombia | ─ | ─ | ─ | Genbank |
| *P. lasalleorum* | *P. leptolophus* | KY627782.1 | MHUAA 8710 | Colombia | ─ | ─ | ─ | Genbank |
| *P. uranobates* | *P. leptolophus* | KY627796.1 | MHUAA 9867 | Colombia | ─ | ─ | ─ | Genbank |
| *P. permixtus* | *P. leptolophus* | DQ195467.1 | CAP 765 | NA | ─ | ─ | ─ | Genbank |
