# Supplementary figures and images for: Unveiling the evolutionary relationships and the high cryptic diversity in Andean rainfrogs (Craugastoridae: Pristimantis myersi group)
Source: PeerJ. 2023 Mar 1;11:e14715. doi: 10.7717/peerj.14715 (PMC9985417; doi:10.7717/peerj.14715)

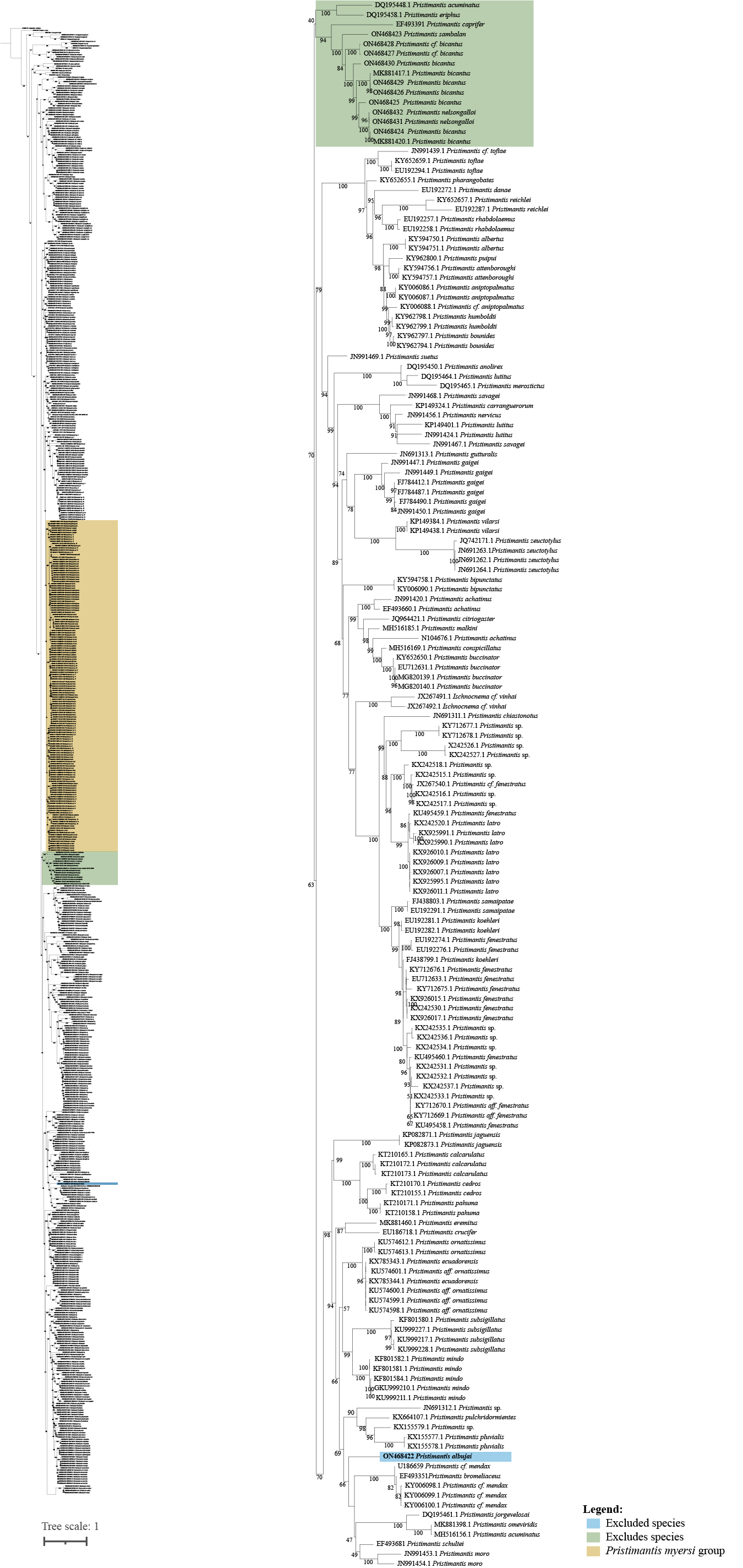

Supplement: Supplemental Information 2 — Node support is expressed in Bootstrap values; missing values indicate support below 70 (bootstrap). Each terminal includes the following information: GenBank code and species name. Highlighted branches indicated new sequences and excluded species of the Pristimantis myersi species group. [file peerj-11-14715-s002.png]

Tree scale: 0.1

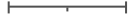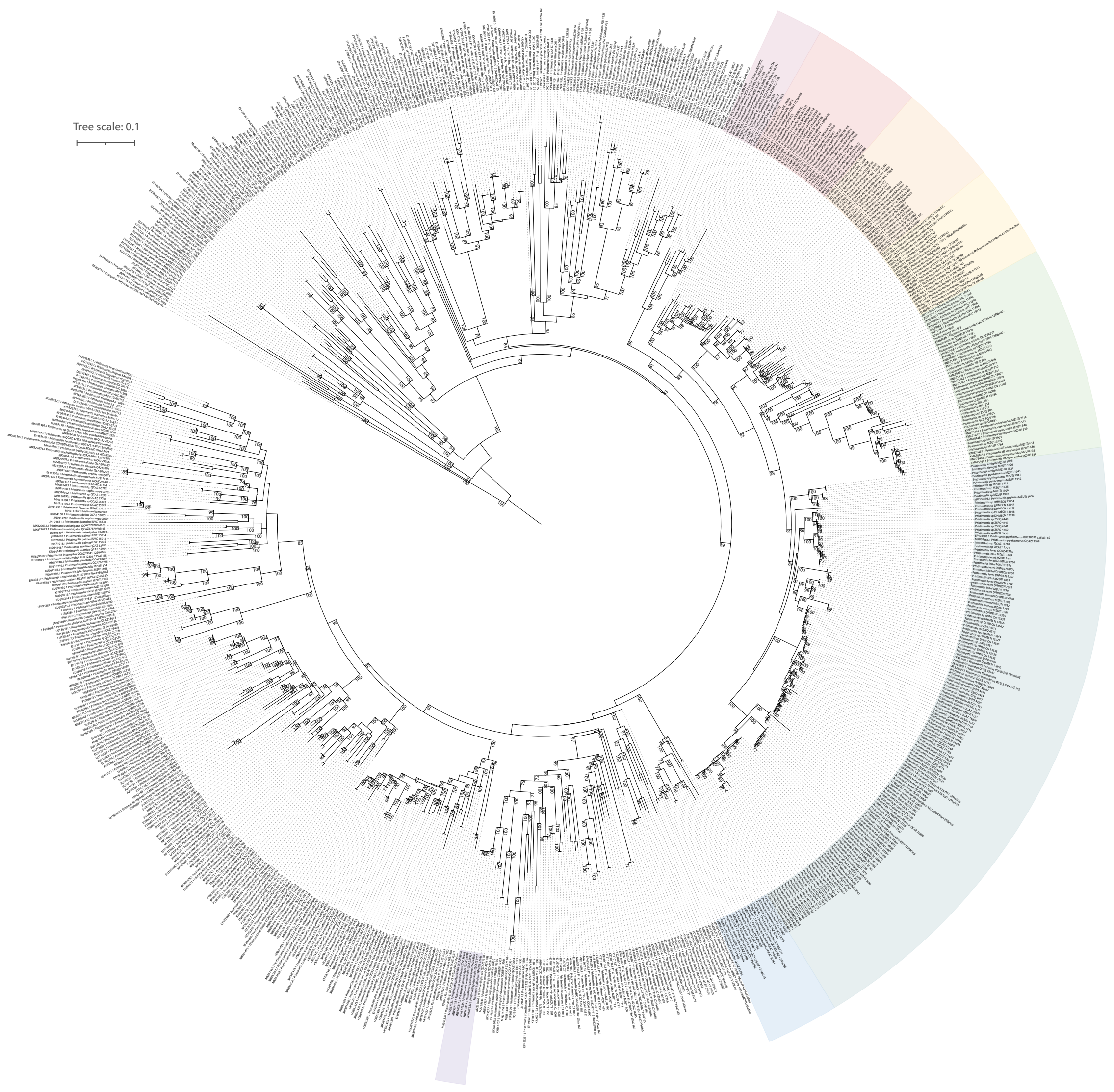

Supplement: Supplemental Information 3 — Node support is expressed in Bootstrap values; missing values indicate support below 70 (bootstrap). Each terminal includes the following information: GenBank code, species name and voucher number. Highlighted branches indicated sequences of Pristimantis myersi group + close relatives clade + excluded species. [file peerj-11-14715-s003.pdf]
