# Supplementary material for: Unveiling the evolutionary relationships and the high cryptic diversity in Andean rainfrogs (Craugastoridae: Pristimantis myersi group)
Source: PeerJ. 2023 Mar 1;11:e14715. doi: 10.7717/peerj.14715 (PMC9985417; doi:10.7717/peerj.14715)
Supplement: Supplemental Information 4 — Values are presented as percent distances calculated from uncorrected p values. [file peerj-11-14715-s004.docx]

**Tables supplemental Information 2.** Genetic distances (mitochondrial 16S) of *Pristimantis myersi group* and *P.* *verecundus* clade and its most closely related congeners. Values are presented as percent distances calculated from uncorrected *p* values.

**Table S1.** Uncorrected genetic distance (16S) *P. jubatus* + *Pristimantis* sp. 1.

|  | **Terminals** | **1** | **2** | **3** | **4** |
| --- | --- | --- | --- | --- | --- |
| **1** | *Pristimantis jubatus*_Colombia_Cali_JN104665_UVC15847 | – |  |  |  |
| **2** | *P_jubatus*_Co_Cali_JN104663_UVC15877 | 0.0 | – |  |  |
| **3** | *Pristimantis* sp. 1_Co_Cauca_JN104680_UVC15942 | 8.3 | 8.3 | – |  |
| **4** | *Pristimantis* sp. 1_Co_Cauca_JN371037_UVC15943 | 8.2 | 8.2 | 0.2 | – |

**Table S2.** Uncorrected genetic distance (16S) species clade *Pristimantis verecundus*.

|  | **Terminals** | **1** | **2** | **3** | **4** | **5** | **6** | **7** | **8** | **9** | **10** | **11** | **12** | **13** | **14** | **15** | **16** | **17** | **18** | **19** | **20** | **21** | **22** | **23** | **24** | **25** | **26** | **27** | **28** | **29** | **30** | **31** | **32** | **33** | **34** | **35** | **36** | **37** | **38** | **39** |
| --- | --- | --- | --- | --- | --- | --- | --- | --- | --- | --- | --- | --- | --- | --- | --- | --- | --- | --- | --- | --- | --- | --- | --- | --- | --- | --- | --- | --- | --- | --- | --- | --- | --- | --- | --- | --- | --- | --- | --- | --- |
| **1** | *Pristimantis* sp. 2_Ec_Cot_Otonga_EF493686_QCAZ12410 | – |  |  |  |  |  |  |  |  |  |  |  |  |  |  |  |  |  |  |  |  |  |  |  |  |  |  |  |  |  |  |  |  |  |  |  |  |  |  |
| **2** | *Pristimantis* sp. 3_Ec_Car_Via_SanLorenzo_JMG675 | 5.0 | – |  |  |  |  |  |  |  |  |  |  |  |  |  |  |  |  |  |  |  |  |  |  |  |  |  |  |  |  |  |  |  |  |  |  |  |  |  |
| **3** | *Pristimantis* sp. 4_Ec_Car_ElPailon_DHMECH13988 | 12.2 | 11.9 | – |  |  |  |  |  |  |  |  |  |  |  |  |  |  |  |  |  |  |  |  |  |  |  |  |  |  |  |  |  |  |  |  |  |  |  |  |
| **4** | *Pristimantis* sp. 5_Ec_Car_Maldonado_DHMECN14985 | 11.3 | 10.4 | 12.0 | – |  |  |  |  |  |  |  |  |  |  |  |  |  |  |  |  |  |  |  |  |  |  |  |  |  |  |  |  |  |  |  |  |  |  |  |
| **5** | *Pristimantis* sp. 5_Ec_Car_Maldonado_DHMECN14858 | 10.7 | 10.4 | 11.8 | 0.0 | – |  |  |  |  |  |  |  |  |  |  |  |  |  |  |  |  |  |  |  |  |  |  |  |  |  |  |  |  |  |  |  |  |  |  |
| **6** | *P.* *celator*_Ec_Car_Maldonado_EF493685_KU177684 | 9.4 | 9.9 | 13.1 | 11.2 | 10.5 | – |  |  |  |  |  |  |  |  |  |  |  |  |  |  |  |  |  |  |  |  |  |  |  |  |  |  |  |  |  |  |  |  |  |
| **7** | *P. mutabilis*_Ec_Pic_Gralarias_KM675457_MZUTI2191 | 10.4 | 10.1 | 13.3 | 11.0 | 10.3 | 9.1 | – |  |  |  |  |  |  |  |  |  |  |  |  |  |  |  |  |  |  |  |  |  |  |  |  |  |  |  |  |  |  |  |  |
| **8** | *P. mutabilis* _Ec_Pic_Gralarias_KM675458_MZUTI2190 | 10.4 | 10.1 | 13.3 | 11.0 | 10.3 | 9.1 | 0.0 | – |  |  |  |  |  |  |  |  |  |  |  |  |  |  |  |  |  |  |  |  |  |  |  |  |  |  |  |  |  |  |  |
| **9** | *P. mutabilis* _Ec_Imb_LosCedros_KM675460_MZUTI912 | 10.8 | 11.0 | 14.4 | 11.9 | 11.4 | 9.5 | 2.4 | 2.4 | – |  |  |  |  |  |  |  |  |  |  |  |  |  |  |  |  |  |  |  |  |  |  |  |  |  |  |  |  |  |  |
| **10** | *P. mutabilis* _Ec_Imb_Manduriacu_ZSFQ504 | 11.0 | 11.1 | 14.3 | 11.2 | 10.9 | 9.5 | 2.5 | 2.5 | 0.8 | – |  |  |  |  |  |  |  |  |  |  |  |  |  |  |  |  |  |  |  |  |  |  |  |  |  |  |  |  |  |
| **11** | *P. mutabilis* _Ec_Imb_Manduriacu_219_027 | 11.0 | 11.0 | 14.2 | 11.2 | 10.9 | 9.5 | 2.5 | 2.5 | 0.8 | 0.0 | – |  |  |  |  |  |  |  |  |  |  |  |  |  |  |  |  |  |  |  |  |  |  |  |  |  |  |  |  |
| **12** | *P. mutabilis* _Ec_Imb_LosCedros_KM675463_MZUTI909 | 11.2 | 10.9 | 14.1 | 11.1 | 10.6 | 9.3 | 2.3 | 2.3 | 0.5 | 0.6 | 0.6 | – |  |  |  |  |  |  |  |  |  |  |  |  |  |  |  |  |  |  |  |  |  |  |  |  |  |  |  |
| **13** | *P. mutabilis* _Ec_Imb_LosCedros_KM675459_MZUTI913 | 11.2 | 11.0 | 14.1 | 11.3 | 10.8 | 9.4 | 2.3 | 2.3 | 0.5 | 0.6 | 0.6 | 0.0 | – |  |  |  |  |  |  |  |  |  |  |  |  |  |  |  |  |  |  |  |  |  |  |  |  |  |  |
| **14** | *P. mutabilis* _Ec_Imb_LosCedros_KM675462_MZUTI910 | 11.3 | 11.2 | 13.9 | 11.3 | 10.9 | 9.2 | 2.4 | 2.4 | 0.6 | 0.7 | 0.7 | 0.1 | 0.1 | – |  |  |  |  |  |  |  |  |  |  |  |  |  |  |  |  |  |  |  |  |  |  |  |  |  |
| **15** | *P. mutabilis* _Ec_Imb_LosCedros_KM675461_MZUTI911 | 11.3 | 11.2 | 13.9 | 11.3 | 10.9 | 9.2 | 2.4 | 2.4 | 0.6 | 0.7 | 0.7 | 0.1 | 0.1 | 0.0 | – |  |  |  |  |  |  |  |  |  |  |  |  |  |  |  |  |  |  |  |  |  |  |  |  |
| **16** | *P. verecundus*_Ec_Car_Dracula_DHMECN15007 | 11.2 | 10.6 | 14.6 | 12.6 | 12.1 | 8.8 | 9.0 | 9.0 | 9.0 | 9.1 | 9.0 | 8.7 | 8.7 | 8.8 | 8.8 | – |  |  |  |  |  |  |  |  |  |  |  |  |  |  |  |  |  |  |  |  |  |  |  |
| **17** | *P. verecundus* _Ec_Car_Dracula_DHMECN15188 | 11.2 | 10.5 | 14.6 | 12.4 | 11.8 | 8.7 | 9.3 | 9.3 | 9.4 | 9.1 | 9.1 | 9.2 | 9.2 | 9.3 | 9.3 | 0.5 | – |  |  |  |  |  |  |  |  |  |  |  |  |  |  |  |  |  |  |  |  |  |  |
| **18** | *P. verecundus* _Ec_Car_Chical_DHMECH12599 | 11.6 | 10.6 | 14.4 | 12.2 | 12.2 | 9.1 | 9.2 | 9.2 | 9.3 | 9.0 | 9.0 | 9.0 | 9.0 | 9.2 | 9.2 | 0.3 | 0.6 | – |  |  |  |  |  |  |  |  |  |  |  |  |  |  |  |  |  |  |  |  |  |
| **19** | *P. verecundus* _Ec_Car_Dracula_DHMECN15189 | 11.4 | 10.6 | 14.4 | 12.6 | 12.0 | 8.9 | 9.3 | 9.3 | 9.5 | 9.0 | 9.0 | 9.3 | 9.3 | 9.4 | 9.4 | 0.3 | 0.5 | 0.0 | – |  |  |  |  |  |  |  |  |  |  |  |  |  |  |  |  |  |  |  |  |
| **20** | *Pristimantis* sp. 6_Ec_Car_ElPailon_DHMECH14006 | 11.3 | 10.8 | 13.4 | 12.9 | 12.7 | 10.5 | 8.9 | 8.9 | 9.1 | 9.1 | 9.1 | 9.1 | 9.1 | 9.3 | 9.3 | 8.5 | 8.6 | 8.1 | 8.1 | – |  |  |  |  |  |  |  |  |  |  |  |  |  |  |  |  |  |  |  |
| **21** | *Pristimantis* sp. 7_Ec_Car_ElPailon_DHMECH13984 | 11.6 | 12.6 | 14.3 | 12.6 | 12.7 | 10.7 | 9.2 | 9.2 | 9.1 | 8.9 | 8.8 | 8.9 | 8.8 | 9.0 | 9.0 | 9.0 | 8.9 | 8.7 | 8.7 | 6.1 | – |  |  |  |  |  |  |  |  |  |  |  |  |  |  |  |  |  |  |
| **22** | *Pristimantis* sp. 8_Ec_Esm_Canande_JMG253 | 16.3 | 15.1 | 16.3 | 16.0 | 16.0 | 11.7 | 9.8 | 9.8 | 10.2 | 10.2 | 10.2 | 9.7 | 9.7 | 10.2 | 10.2 | 8.7 | 8.7 | 8.7 | 8.7 | 7.8 | 5.8 | – |  |  |  |  |  |  |  |  |  |  |  |  |  |  |  |  |  |
| **23** | *Pristimantis* sp. 8_Ec_Esm_Canande_JMG255 | 12.3 | 11.6 | 14.8 | 12.7 | 12.7 | 10.8 | 7.9 | 7.9 | 8.1 | 8.1 | 8.1 | 7.9 | 7.9 | 8.1 | 8.1 | 7.6 | 7.4 | 7.6 | 7.6 | 6.4 | 4.9 | 1.0 | – |  |  |  |  |  |  |  |  |  |  |  |  |  |  |  |  |
| **24** | *Pristimantis* sp. 9_Ec_Imb_Manduriacu_ZSFQ503 | 10.7 | 10.4 | 13.2 | 12.2 | 12.3 | 9.4 | 8.0 | 8.0 | 8.3 | 8.3 | 8.3 | 8.3 | 8.3 | 8.4 | 8.4 | 6.5 | 6.6 | 6.3 | 6.3 | 3.7 | 4.8 | 6.8 | 5.1 | – |  |  |  |  |  |  |  |  |  |  |  |  |  |  |  |
| **25** | *Pristimantis* sp. 9_Ec_Imb_Manduriacu_219_011 | 10.6 | 10.3 | 13.1 | 12.0 | 12.1 | 9.2 | 7.9 | 7.9 | 8.1 | 8.1 | 8.1 | 8.1 | 8.1 | 8.3 | 8.3 | 6.8 | 6.8 | 6.6 | 6.6 | 3.6 | 4.7 | 6.3 | 4.9 | 0.1 | – |  |  |  |  |  |  |  |  |  |  |  |  |  |  |
| **26** | *Pristimantis* sp. 9_Ec_Imb_Manduriacu_ZSFQ_0506 | 10.7 | 10.3 | 13.2 | 12.2 | 12.3 | 9.2 | 8.0 | 8.0 | 8.3 | 8.3 | 8.3 | 8.3 | 8.3 | 8.4 | 8.4 | 6.7 | 6.7 | 6.4 | 6.4 | 3.7 | 4.8 | 6.8 | 5.1 | 0.3 | 0.1 | – |  |  |  |  |  |  |  |  |  |  |  |  |  |
| **27** | *Pristimantis* sp. 9_Ec_Imb_Manduriacu_ZSFQ0505 | 10.7 | 10.3 | 13.2 | 12.2 | 12.3 | 9.2 | 8.3 | 8.3 | 8.4 | 8.4 | 8.4 | 8.4 | 8.4 | 8.5 | 8.5 | 6.8 | 7.0 | 6.7 | 6.7 | 3.7 | 4.8 | 6.8 | 5.1 | 0.3 | 0.1 | 0.0 | – |  |  |  |  |  |  |  |  |  |  |  |  |
| **28** | *Pristimantis* sp. 9_Ec_Imb_Manduriacu_ZSFQ0485 | 10.9 | 10.4 | 13.4 | 12.3 | 12.4 | 9.4 | 8.2 | 8.2 | 8.4 | 8.4 | 8.4 | 8.4 | 8.4 | 8.6 | 8.6 | 6.8 | 6.9 | 6.6 | 6.6 | 3.8 | 5.0 | 6.8 | 5.1 | 0.4 | 0.3 | 0.1 | 0.1 | – |  |  |  |  |  |  |  |  |  |  |  |
| **29** | *Pristimantis* sp. 10_Ec_Pic_Mindo_KM675445_MZUTI2114 | 11.2 | 11.8 | 13.6 | 11.8 | 11.4 | 9.3 | 7.9 | 7.9 | 7.8 | 8.7 | 8.7 | 7.9 | 7.8 | 8.0 | 8.0 | 6.6 | 6.8 | 6.8 | 6.4 | 4.7 | 5.9 | 9.2 | 6.9 | 3.1 | 2.9 | 3.1 | 3.1 | 3.3 | – |  |  |  |  |  |  |  |  |  |  |
| **30** | *Pristimantis* sp. 10_Ec_Pic_Mindo_KM675446_MZUTI541 | 11.3 | 11.8 | 13.6 | 11.8 | 11.4 | 9.3 | 7.8 | 7.8 | 7.7 | 8.7 | 8.7 | 7.8 | 7.7 | 7.9 | 7.9 | 6.6 | 6.8 | 6.8 | 6.4 | 4.7 | 5.9 | 9.2 | 6.9 | 3.1 | 2.9 | 3.1 | 3.1 | 3.3 | 0.0 | – |  |  |  |  |  |  |  |  |  |
| **31** | *Pristimantis* sp. 10_Ec_Pic_Mindo_KM675447_MZUTI540 | 13.0 | 12.6 | 14.2 | 12.6 | 12.6 | 11.0 | 8.8 | 8.8 | 8.7 | 8.9 | 8.9 | 8.7 | 8.7 | 8.9 | 8.9 | 7.6 | 7.8 | 7.4 | 7.4 | 4.8 | 5.8 | 9.2 | 6.7 | 3.1 | 2.9 | 3.1 | 3.1 | 3.3 | 0.0 | 0.0 | – |  |  |  |  |  |  |  |  |
| **32** | *Pristimantis* sp. 10_Ec_Pic_Mindo_KM675448_MZUTI539 | 11.2 | 11.3 | 13.9 | 11.4 | 10.9 | 9.2 | 7.5 | 7.5 | 7.3 | 8.2 | 8.1 | 7.3 | 7.3 | 7.4 | 7.4 | 6.6 | 6.6 | 6.7 | 6.4 | 4.6 | 5.8 | 9.2 | 6.9 | 3.2 | 3.0 | 3.2 | 3.2 | 3.4 | 0.0 | 0.0 | 0.0 | – |  |  |  |  |  |  |  |
| **33** | *Pristimantis* sp. 10_Ec_Pic_Mashpi_MZUTI3903 | 11.4 | 11.4 | 13.2 | 12.6 | 12.5 | 10.2 | 9.0 | 9.0 | 8.8 | 9.1 | 9.1 | 9.1 | 9.1 | 9.2 | 9.2 | 7.9 | 8.1 | 7.8 | 7.8 | 4.7 | 5.7 | 9.7 | 7.1 | 2.7 | 2.7 | 2.7 | 2.8 | 2.8 | 0.7 | 0.7 | 0.8 | 0.7 | – |  |  |  |  |  |  |
| **34** | *Pristimantis* sp. 10_Ec_Pic_Mashpi_MZUTI3764 | 11.7 | 11.7 | 13.6 | 12.9 | 12.8 | 10.4 | 9.4 | 9.4 | 9.2 | 9.5 | 9.5 | 9.5 | 9.5 | 9.6 | 9.6 | 8.2 | 8.4 | 8.1 | 8.1 | 5.0 | 6.0 | 10.2 | 7.4 | 3.0 | 3.0 | 3.0 | 3.1 | 3.1 | 1.0 | 1.0 | 1.2 | 0.9 | 0.3 | – |  |  |  |  |  |
| **35** | *Pristimantis* sp. 10_Ec_Pic_Mashpi_MZUTI3922 | 11.6 | 11.5 | 13.4 | 12.7 | 12.7 | 10.2 | 9.2 | 9.2 | 9.1 | 9.4 | 9.4 | 9.4 | 9.3 | 9.5 | 9.5 | 8.1 | 8.3 | 7.9 | 7.9 | 4.8 | 5.8 | 9.7 | 7.1 | 2.8 | 2.8 | 2.8 | 3.0 | 3.0 | 0.9 | 0.9 | 1.0 | 0.7 | 0.1 | 0.1 | – |  |  |  |  |
| **36** | *Pristimantis* sp. 10_Ec-Pic_Chontilla_KM675465_MZUTI635 | 10.9 | 11.5 | 13.4 | 12.8 | 12.1 | 9.7 | 8.6 | 8.6 | 8.8 | 9.0 | 8.9 | 9.1 | 9.1 | 9.2 | 9.2 | 7.6 | 8.2 | 7.8 | 7.9 | 4.6 | 5.5 | 9.2 | 6.9 | 2.7 | 2.6 | 2.7 | 2.7 | 2.8 | 0.8 | 0.8 | 1.0 | 0.8 | 0.4 | 0.7 | 0.6 | – |  |  |  |
| **37** | *Pristimantis* sp. 10_Ec_Pic_Chontilla_KM675466_MZUTI634 | 10.9 | 11.5 | 13.4 | 12.8 | 12.1 | 9.7 | 8.6 | 8.6 | 8.8 | 9.0 | 8.9 | 9.1 | 9.1 | 9.2 | 9.2 | 7.6 | 8.2 | 7.8 | 7.9 | 4.6 | 5.5 | 9.2 | 6.9 | 2.7 | 2.6 | 2.7 | 2.7 | 2.8 | 0.8 | 0.8 | 1.0 | 0.8 | 0.4 | 0.7 | 0.6 | 0.0 | – |  |  |
| **38** | *Pristimantis* sp. 10_Ec_Pic_Chontilla_KM675464_MZUTI636 | 11.4 | 11.5 | 13.4 | 12.8 | 12.1 | 9.8 | 8.6 | 8.6 | 8.9 | 9.0 | 8.9 | 9.2 | 9.2 | 9.3 | 9.3 | 7.6 | 8.2 | 7.8 | 7.9 | 4.6 | 5.5 | 9.2 | 6.9 | 2.7 | 2.6 | 2.7 | 2.7 | 2.8 | 0.8 | 0.8 | 1.0 | 0.8 | 0.4 | 0.7 | 0.6 | 0.0 | 0.0 | – |  |
| **39** | *Pristimantis* sp. 10_Ec_Pic_Chontilla_KM675467_MZUTI633 | 11.5 | 11.5 | 13.4 | 12.8 | 12.1 | 9.8 | 8.5 | 8.5 | 8.9 | 9.0 | 9.0 | 9.2 | 9.2 | 9.3 | 9.3 | 7.5 | 7.9 | 7.5 | 7.7 | 4.6 | 5.5 | 9.2 | 6.9 | 2.7 | 2.6 | 2.7 | 2.7 | 2.8 | 0.8 | 0.8 | 1.0 | 0.8 | 0.3 | 0.6 | 0.4 | 0.0 | 0.0 | 0.0 | – |

**Table S3.** Uncorrected genetic distance (16S), subclade 1 of *Pristimantis myersi* group.

|  | **Terminals** | **1** | **2** | **3** | **4** | **5** | **6** | **7** | **8** | **9** | **10** |
| --- | --- | --- | --- | --- | --- | --- | --- | --- | --- | --- | --- |
| **1** | *Pristimantis sirnigeli*_Ec_Pic_Verdecocha_MZUTI1825 | – |  |  |  |  |  |  |  |  |  |
| **2** | *P. sirnigeli*_Ec_Pic_Verdecocha_MZUTI1826 | 0.0 | – |  |  |  |  |  |  |  |  |
| **3** | *P. sirnigeli* _Ec_Pic_Verdecocha_MZUTI1827 | 0.0 | 0.0 | – |  |  |  |  |  |  |  |
| **4** | *P. pyrrhomerus*_Ec_Cot_Pilalo_MZUTI1941 | 3.5 | 3.5 | 3.5 | – |  |  |  |  |  |  |
| **5** | *P. pyrrhomerus* _Ec_Cot_Pilalo_MZUTI1942 | 3.7 | 3.7 | 3.7 | 0.0 | – |  |  |  |  |  |
| **6** | *P. pyrrhomerus* _Ec_Cot_Pilalo_MZUTI1943 | 3.6 | 3.6 | 3.6 | 0.0 | 0.0 | – |  |  |  |  |
| **7** | *Pristimantis* sp. 11_Ec_Cot_Sigchos_MZUTI1925 | 2.8 | 2.7 | 2.7 | 2.3 | 2.3 | 2.2 | – |  |  |  |
| **8** | *Pristimantis* sp. 11_Ec_Cot_Sigchos_MZUTI1926 | 2.8 | 2.7 | 2.7 | 2.3 | 2.3 | 2.2 | 0.0 | – |  |  |
| **9** | *Pristimantis* sp. 11_Ec_Cot_Sigchos_MZUTI1927 | 2.8 | 2.7 | 2.7 | 2.3 | 2.3 | 2.2 | 0.0 | 0.0 | – |  |
| **10** | *Pristimantis* sp. 11_Ec_Cot_Sigchos_MZUTI1928 | 2.8 | 2.7 | 2.7 | 2.3 | 2.3 | 2.2 | 0.0 | 0.0 | 0.0 | – |

**Table S4.** Uncorrected genetic distance (16S), subclade 2 of *Pristimantis myersi* group.

|  | **Terminals** | **1** | **2** | **3** | **4** | **5** | **6** | **7** | **8** | **9** | **10** |
| --- | --- | --- | --- | --- | --- | --- | --- | --- | --- | --- | --- |
| **1** | *Pristimantis* sp.12_Ec_Car_VirgenNegra_DHMECN13354 | – |  |  |  |  |  |  |  |  |  |
| **2** | *Pristimantis* sp.12_Ec_Car_VirgenNegra_DHMECN13347 | 0.1 | – |  |  |  |  |  |  |  |  |
| **3** | *Pristimantis* sp.12_Ec_Car_LaEsperanza_DHMECN13649 | 0.2 | 0.1 | – |  |  |  |  |  |  |  |
| **4** | *Pristimantis* sp.12_Ec_Car_LaEsperanza_DHMECN13648 | 0.3 | 0.1 | 0.0 | – |  |  |  |  |  |  |
| **5** | *Pristimantis* sp.12_Ec_Car_Moran_DHMECN13339 | 1.3 | 1.2 | 1.3 | 1.3 | – |  |  |  |  |  |
| **6** | *Pristimantis* sp.12_Ec_Car_Moran_DBR406 | 1.8 | 1.6 | 1.8 | 1.8 | 0.5 | – |  |  |  |  |
| **7** | *Pristimantis* sp.12_Ec_Car_TufinoMaldonado_DBR264 | 1.5 | 1.4 | 1.5 | 1.6 | 0.3 | 0.4 | – |  |  |  |
| **8** | *Pristimantis* sp.12_Ec_Car_TufinoMaldonado_DBR269 | 1.4 | 1.3 | 1.4 | 1.5 | 0.1 | 0.3 | 0.1 | – |  |  |
| **9** | *Pristimantis* sp.12_Ec_Car_TufinoMaldonado_DBR266 | 1.4 | 1.3 | 1.4 | 1.5 | 0.1 | 0.3 | 0.1 | 0.0 | – |  |
| **10** | *Pristimantis* sp.12_Ec_Car_TufinoMaldonado_DBR263 | 1.7 | 1.6 | 1.6 | 1.7 | 0.4 | 0.6 | 0.4 | 0.3 | 0.3 | – |

**Table S5.** Uncorrected genetic distance (16S), subclade 3 of *Pristimantis myersi* group.

|  | **Terminals** | **1** | **2** | **3** | **4** | **5** | **6** | **7** | **8** | **9** | **10** | **11** | **12** | **13** | **14** | **15** | **16** |
| --- | --- | --- | --- | --- | --- | --- | --- | --- | --- | --- | --- | --- | --- | --- | --- | --- | --- |
| **1** | *Pristimantis* sp. 13_Ec_Bol_BP_Totoras_EF493683_KU218030 | – |  |  |  |  |  |  |  |  |  |  |  |  |  |  |  |
| **2** | *Pristimantis* sp. 13_Ec_Bol_BP_Totoras_QCAZ13771 | 0.2 | – |  |  |  |  |  |  |  |  |  |  |  |  |  |  |
| **3** | *Pristimantis* sp. 13_Ec_Bol_BP_Totoras_QCAZ13769 | 0.2 | 0.0 | – |  |  |  |  |  |  |  |  |  |  |  |  |  |
| **4** | *P. leoni*_Ec_Imb_LagunaMojanda_QCAZ42125 | 1.5 | 1.3 | 1.3 | – |  |  |  |  |  |  |  |  |  |  |  |  |
| **5** | *P. leoni* _Ec_Imb_LagunaMojanda_MZUTI1809 | 1.6 | 1.3 | 1.3 | 0.0 | – |  |  |  |  |  |  |  |  |  |  |  |
| **6** | *P. leoni* _Ec_Imb_LagunaMojanda_MZUTI1811 | 1.6 | 1.3 | 1.3 | 0.0 | 0.0 | – |  |  |  |  |  |  |  |  |  |  |
| **7** | *P. leoni* _Ec_Pic_Yanacocha_DHMECN7285 | 1.9 | 1.8 | 1.8 | 0.4 | 0.4 | 0.4 | – |  |  |  |  |  |  |  |  |  |
| **8** | *P. leoni* _Ec_Pic_Yanacocha_DHMECN7287 | 1.9 | 1.7 | 1.7 | 0.5 | 0.5 | 0.5 | 0.0 | – |  |  |  |  |  |  |  |  |
| **9** | *P. leoni* _Ec_Pic_Verdecocha_MZUTI1799 | 2.1 | 1.9 | 1.9 | 0.5 | 0.6 | 0.6 | 0.0 | 0.0 | – |  |  |  |  |  |  |  |
| **10** | *P. leoni* _Ec_Pic_Verdecocha_MZUTI1818 | 1.9 | 1.9 | 1.8 | 0.5 | 0.6 | 0.6 | 0.0 | 0.0 | 0.1 | – |  |  |  |  |  |  |
| **11** | *P. leoni* _Ec_Pic_Verdecocha_MZUTI1819 | 1.9 | 1.9 | 1.8 | 0.5 | 0.6 | 0.6 | 0.0 | 0.0 | 0.1 | 0.0 | – |  |  |  |  |  |
| **12** | *P. leoni* _Ec_Pic_ValleDelToaza_DHMECN8756 | 1.8 | 1.9 | 1.8 | 0.7 | 0.8 | 0.8 | 0.1 | 0.1 | 0.4 | 0.2 | 0.2 | – |  |  |  |  |
| **13** | *P. leoni* _Ec_Pic_ValleDelToaza_DHMECN8762 | 1.9 | 1.9 | 1.8 | 0.5 | 0.6 | 0.6 | 0.0 | 0.0 | 0.1 | 0.0 | 0.0 | 0.2 | – |  |  |  |
| **14** | *P. leoni* _Ec_Pic_ValleDelToaza_DHMECN8759 | 2.2 | 2.0 | 2.0 | 0.7 | 0.7 | 0.7 | 0.1 | 0.2 | 0.4 | 0.3 | 0.3 | 0.6 | 0.3 | – |  |  |
| **15** | *P. leoni* _Ec_Pic_ValleDelToaza_DHMECN8757 | 2.1 | 2.1 | 2.1 | 0.7 | 0.8 | 0.8 | 0.1 | 0.2 | 0.4 | 0.2 | 0.2 | 0.5 | 0.2 | 0.1 | – |  |
| **16** | *P. leoni* _Ec_Pic_ValleDelToaza_DHMECN8760 | 2.1 | 2.1 | 2.1 | 0.7 | 0.8 | 0.8 | 0.1 | 0.2 | 0.4 | 0.2 | 0.2 | 0.5 | 0.2 | 0.1 | 0.0 | – |

**Table S6.** Uncorrected genetic distance (16S), subclade 4 of *Pristimantis myersi* group.

|  | **Terminals** | **1** | **2** | **3** | **4** | **5** | **6** | **7** | **8** | **9** | **10** | **11** | **12** | **13** | **14** | **15** | **16** | **17** | **18** | **19** | **20** | **21** | **22** | **23** | **24** | **25** | **26** | **27** | **28** | **29** | **30** |
| --- | --- | --- | --- | --- | --- | --- | --- | --- | --- | --- | --- | --- | --- | --- | --- | --- | --- | --- | --- | --- | --- | --- | --- | --- | --- | --- | --- | --- | --- | --- | --- |
| **1** | *Pristimantis munozi*_Ec_Pic_LaVictoria_DHMECN4938 | – |  |  |  |  |  |  |  |  |  |  |  |  |  |  |  |  |  |  |  |  |  |  |  |  |  |  |  |  |  |
| **2** | *P. munozi*_Ec_Pic_Verdecocha_MZUTI1779 | 0.3 | – |  |  |  |  |  |  |  |  |  |  |  |  |  |  |  |  |  |  |  |  |  |  |  |  |  |  |  |  |
| **3** | *P. munozi* _Ec_Pic_Verdecocha_MZUTI1782 | 0.3 | 0.0 | – |  |  |  |  |  |  |  |  |  |  |  |  |  |  |  |  |  |  |  |  |  |  |  |  |  |  |  |
| **4** | *P. munozi* _Ec_Pic_Verdecocha_MZUTI1783 | 0.6 | 0.4 | 0.4 | – |  |  |  |  |  |  |  |  |  |  |  |  |  |  |  |  |  |  |  |  |  |  |  |  |  |  |
| **5** | *P. munozi* _Ec_Pic_Verdecocha_MZUTI1784 | 0.3 | 0.0 | 0.0 | 0.4 | – |  |  |  |  |  |  |  |  |  |  |  |  |  |  |  |  |  |  |  |  |  |  |  |  |  |
| **6** | *Pristimantis* sp. 14_Ec_Car_LaBreta_a_DHMECN13329 | 5.9 | 5.6 | 5.6 | 5.8 | 5.6 | – |  |  |  |  |  |  |  |  |  |  |  |  |  |  |  |  |  |  |  |  |  |  |  |  |
| **7** | *Pristimantis* sp. 14_Car_VirgenNegra_DHMECN13355_DFMMyersijun | 5.9 | 5.6 | 5.6 | 5.7 | 5.6 | 0.1 | – |  |  |  |  |  |  |  |  |  |  |  |  |  |  |  |  |  |  |  |  |  |  |  |
| **8** | *Pristimantis* sp. 14_Car_VirgenNegra_DHMECN13356_DFMMyersijun | 5.9 | 5.5 | 5.5 | 5.6 | 5.5 | 0.1 | 0.0 | – |  |  |  |  |  |  |  |  |  |  |  |  |  |  |  |  |  |  |  |  |  |  |
| **9** | *Pristimantis* sp. 14_Ec_Car_LaBreta_a_DHMECN13327 | 4.4 | 4.2 | 4.2 | 4.2 | 4.2 | 1.9 | 2.0 | 2.0 | – |  |  |  |  |  |  |  |  |  |  |  |  |  |  |  |  |  |  |  |  |  |
| **10** | *Pristimantis* sp. 14_Car_LaEsperanza_DHMECN13645_DFMMyersijun | 5.5 | 5.3 | 5.3 | 5.4 | 5.3 | 2.6 | 2.8 | 2.7 | 0.0 | – |  |  |  |  |  |  |  |  |  |  |  |  |  |  |  |  |  |  |  |  |
| **11** | *Pristimantis* sp. 14_Car_LaEsperanza_DHMECN13644_DFMMyersijun | 5.4 | 5.1 | 5.1 | 5.3 | 5.1 | 2.5 | 2.6 | 2.6 | 0.0 | 0.1 | – |  |  |  |  |  |  |  |  |  |  |  |  |  |  |  |  |  |  |  |
| **12** | *Pristimantis* sp. 14_Car_LaEsperanza_DHMECN13642_DFMMyersijun | 5.4 | 5.2 | 5.2 | 5.3 | 5.2 | 2.5 | 2.6 | 2.5 | 0.0 | 0.1 | 0.0 | – |  |  |  |  |  |  |  |  |  |  |  |  |  |  |  |  |  |  |
| **13** | *Pristimantis* sp. 14_Car_DBR375_Myersijun | 6.0 | 5.9 | 5.9 | 6.0 | 5.9 | 2.6 | 2.8 | 2.8 | 0.2 | 0.3 | 0.2 | 0.2 | – |  |  |  |  |  |  |  |  |  |  |  |  |  |  |  |  |  |
| **14** | *Pristimantis* sp. 14_Car_DBR377_Myersijun | 6.2 | 6.1 | 6.1 | 6.2 | 6.1 | 2.7 | 2.9 | 2.9 | 0.0 | 0.2 | 0.0 | 0.0 | 0.2 | – |  |  |  |  |  |  |  |  |  |  |  |  |  |  |  |  |
| **15** | *Pristimantis* sp. 15_Ec_Car_SanFranciscoPioter_DBR360 | 8.4 | 8.0 | 8.0 | 8.2 | 8.0 | 6.3 | 6.4 | 6.4 | 4.7 | 6.3 | 6.1 | 6.1 | 6.2 | 6.1 | – |  |  |  |  |  |  |  |  |  |  |  |  |  |  |  |
| **16** | *Pristimantis* sp. 15_Ec_Car_SanFranciscoPioter_DHMECN_13633 | 7.8 | 7.5 | 7.5 | 7.5 | 7.5 | 6.3 | 6.5 | 6.5 | 4.9 | 5.7 | 5.7 | 5.7 | 6.5 | 6.5 | 0.0 | – |  |  |  |  |  |  |  |  |  |  |  |  |  |  |
| **17** | *Pristimantis* sp. 15_Ec_Car_SanFranciscoPioter_DHMECN13634 | 7.6 | 7.2 | 7.2 | 7.3 | 7.2 | 5.9 | 6.0 | 6.0 | 4.8 | 5.9 | 5.8 | 5.8 | 6.6 | 6.7 | 0.0 | 0.0 | – |  |  |  |  |  |  |  |  |  |  |  |  |  |
| **18** | *Pristimantis* sp. 15_Ec_Car_SanFranciscoPioter_DHMECN13635 | 8.0 | 7.3 | 7.3 | 7.4 | 7.3 | 6.3 | 6.4 | 6.3 | 5.2 | 6.2 | 6.0 | 6.0 | 7.1 | 7.2 | 0.5 | 0.5 | 0.4 | – |  |  |  |  |  |  |  |  |  |  |  |  |
| **19** | *P. ocreatus* _Ec_Imb_LagunaMojanda_QCAZ42111 | 6.5 | 6.1 | 6.1 | 6.3 | 6.1 | 4.5 | 4.8 | 4.7 | 2.9 | 3.5 | 3.4 | 3.3 | 4.3 | 4.0 | 5.9 | 5.0 | 5.1 | 5.4 | – |  |  |  |  |  |  |  |  |  |  |  |
| **20** | *P. ocreatus* _JX564889 | 6.5 | 6.2 | 6.1 | 6.3 | 6.7 | 4.4 | 4.5 | 4.5 | 2.9 | 3.5 | 3.4 | 3.4 | 4.3 | 4.0 | 5.9 | 5.0 | 5.1 | 5.4 | 0.0 | – |  |  |  |  |  |  |  |  |  |  |
| **21** | *P. ocreatus* _Ec_Car_Moran_DBR412 | 7.8 | 7.5 | 7.5 | 7.7 | 7.5 | 4.9 | 5.1 | 5.1 | 3.7 | 4.1 | 4.0 | 4.0 | 4.2 | 3.8 | 6.5 | 6.0 | 6.3 | 6.8 | 0.8 | 0.8 | – |  |  |  |  |  |  |  |  |  |
| **22** | *P. ocreatus* _Ec_Car_ViaTulcanMaldonado_QCAZ43161 | 6.4 | 6.1 | 6.0 | 6.3 | 6.6 | 4.5 | 4.8 | 4.7 | 2.9 | 3.5 | 3.4 | 3.3 | 4.3 | 4.0 | 5.9 | 5.0 | 5.1 | 5.4 | 0.0 | 0.0 | 0.8 | – |  |  |  |  |  |  |  |  |
| **23** | *P. ocreatus* _Ec_Car_ViaTulcanMaldonado_QCAZ43162 | 6.4 | 6.1 | 6.0 | 6.3 | 6.4 | 4.5 | 4.8 | 4.7 | 2.9 | 3.5 | 3.4 | 3.3 | 4.3 | 4.0 | 5.9 | 5.0 | 5.1 | 5.4 | 0.0 | 0.0 | 0.8 | 0.0 | – |  |  |  |  |  |  |  |
| **24** | *P. ocreatus* _Ec_Car_W_Tulcan_EF493682_KU208508 | 6.6 | 6.3 | 6.2 | 6.4 | 6.8 | 4.7 | 4.8 | 4.7 | 3.1 | 3.6 | 3.5 | 3.5 | 4.5 | 4.2 | 5.9 | 5.1 | 5.2 | 5.5 | 0.1 | 0.1 | 0.9 | 0.1 | 0.1 | – |  |  |  |  |  |  |
| **25** | *P. ocreatus* _Ec_Car_AguasEdiondas_DHMECN13650 | 6.8 | 6.4 | 6.4 | 6.5 | 6.4 | 4.8 | 5.0 | 4.9 | 3.1 | 3.8 | 3.6 | 3.6 | 4.6 | 4.3 | 6.1 | 5.3 | 5.4 | 5.7 | 0.2 | 0.2 | 1.1 | 0.2 | 0.2 | 0.1 | – |  |  |  |  |  |
| **26** | *P. ocreatus* _Ec_Car_AguasEdiondas_DHMECN13669 | 6.5 | 6.1 | 6.1 | 6.4 | 6.1 | 4.5 | 4.7 | 4.7 | 3.1 | 3.7 | 3.5 | 3.5 | 4.3 | 4.0 | 5.9 | 5.0 | 5.1 | 5.5 | 0.0 | 0.0 | 0.8 | 0.0 | 0.0 | 0.1 | 0.3 | – |  |  |  |  |
| **27** | *P. ocreatus* _Ec_Car_Potrerillos_DBR270 | 6.8 | 6.4 | 6.4 | 6.4 | 6.4 | 4.7 | 4.8 | 4.7 | 3.1 | 3.8 | 3.7 | 3.6 | 4.6 | 4.3 | 6.3 | 5.3 | 5.4 | 5.7 | 0.3 | 0.3 | 1.1 | 0.3 | 0.3 | 0.4 | 0.5 | 0.3 | – |  |  |  |
| **28** | *P. ocreatus* _Ec_Car_Potrerillos_DBR271 | 6.9 | 6.5 | 6.5 | 6.5 | 6.5 | 4.9 | 5.0 | 4.9 | 3.2 | 4.1 | 4.0 | 4.0 | 5.0 | 4.7 | 6.0 | 5.4 | 5.4 | 5.6 | 0.5 | 0.5 | 1.4 | 0.5 | 0.5 | 0.6 | 0.8 | 0.5 | 0.3 | – |  |  |
| **29** | *P. ocreatus* _Ec_Car_Potrerillos_DBR272 | 6.6 | 6.3 | 6.3 | 6.3 | 6.3 | 4.6 | 4.7 | 4.6 | 2.9 | 3.6 | 3.5 | 3.5 | 4.5 | 4.2 | 6.1 | 5.1 | 5.2 | 5.5 | 0.1 | 0.1 | 0.9 | 0.1 | 0.1 | 0.3 | 0.4 | 0.1 | 0.1 | 0.4 | – |  |
| **30** | *P. ocreatus*_Ec_Car_Potrerillos_DBR273 | 6.8 | 6.2 | 6.2 | 6.4 | 6.2 | 4.8 | 4.9 | 4.8 | 3.3 | 4.0 | 3.9 | 3.9 | 4.8 | 4.5 | 6.3 | 5.4 | 5.5 | 5.7 | 0.4 | 0.4 | 1.2 | 0.4 | 0.4 | 0.5 | 0.6 | 0.4 | 0.1 | 0.4 | 0.3 | – |

**Table S7.** Uncorrected genetic distance (16S), subclade 5 of *Pristimantis myersi* group.

|  | **Terminals** | **1** | **2** | **3** | **4** | **5** | **6** | **7** | **8** | **9** | **10** | **11** | **12** | **13** | **14** | **15** | **16** | **17** | **18** | **19** | **20** | **21** | **22** | **23** | **24** | **25** | **26** | **27** | **28** | **29** | **30** | **31** | **32** | **33** | **34** | **35** |  |
| --- | --- | --- | --- | --- | --- | --- | --- | --- | --- | --- | --- | --- | --- | --- | --- | --- | --- | --- | --- | --- | --- | --- | --- | --- | --- | --- | --- | --- | --- | --- | --- | --- | --- | --- | --- | --- | --- |
| **1** | *Pristimantis gladiator*_Ec_Nap_Papallacta_MZUTI1117 | – |  |  |  |  |  |  |  |  |  |  |  |  |  |  |  |  |  |  |  |  |  |  |  |  |  |  |  |  |  |  |  |  |  |  |  |
| **2** | *P. gladiator*_Ec_Nap_Papallacta_MZUTI1118 | 0.0 | – |  |  |  |  |  |  |  |  |  |  |  |  |  |  |  |  |  |  |  |  |  |  |  |  |  |  |  |  |  |  |  |  |  |  |
| **3** | *P. gladiator* _Ec_Nap_Papallacta_MZUTI1122 | 0.0 | 0.0 | – |  |  |  |  |  |  |  |  |  |  |  |  |  |  |  |  |  |  |  |  |  |  |  |  |  |  |  |  |  |  |  |  |  |
| **4** | *P. gladiator* _Ec_Nap_Papallacta_MZUTI1123 | 0.2 | 0.2 | 0.2 | – |  |  |  |  |  |  |  |  |  |  |  |  |  |  |  |  |  |  |  |  |  |  |  |  |  |  |  |  |  |  |  |  |
| **5** | *P. gladiator* _Ec_Nap_Papallacta_MZUTI_1131 | 0.1 | 0.1 | 0.1 | 0.3 | – |  |  |  |  |  |  |  |  |  |  |  |  |  |  |  |  |  |  |  |  |  |  |  |  |  |  |  |  |  |  |  |
| **6** | *P. gladiator* _Ec_Nap_Papallacta_MZUTI1214 | 0.0 | 0.0 | 0.0 | 0.2 | 0.1 | – |  |  |  |  |  |  |  |  |  |  |  |  |  |  |  |  |  |  |  |  |  |  |  |  |  |  |  |  |  |  |
| **7** | *P. gladiator* _Ec_Nap_GuangoLodge_QCAZ18874 | 0.1 | 0.1 | 0.1 | 0.4 | 0.2 | 0.1 | – |  |  |  |  |  |  |  |  |  |  |  |  |  |  |  |  |  |  |  |  |  |  |  |  |  |  |  |  |  |
| **8** | *P. gladiator* _Ec_Nap_GuangoLodge_QCAZ18875 | 0.3 | 0.3 | 0.3 | 0.3 | 0.4 | 0.3 | 0.3 | – |  |  |  |  |  |  |  |  |  |  |  |  |  |  |  |  |  |  |  |  |  |  |  |  |  |  |  |  |
| **9** | *Pristimantis* sp. 16_Ec_TungurahuaChamana_DHMECN14701 | 1.8 | 1.8 | 1.8 | 2.0 | 1.7 | 1.8 | 1.8 | 2.2 | – |  |  |  |  |  |  |  |  |  |  |  |  |  |  |  |  |  |  |  |  |  |  |  |  |  |  |  |
| **10** | *Pristimantis* sp. 16_Ec_TungurahuaChamana_DHMECN14702 | 1.9 | 1.9 | 1.9 | 2.2 | 1.8 | 1.9 | 1.9 | 2.3 | 0.0 | – |  |  |  |  |  |  |  |  |  |  |  |  |  |  |  |  |  |  |  |  |  |  |  |  |  |  |
| **11** | *Pristimantis* sp. 16_Ec_TungurahuaCandelaria_DHMECN15824 | 1.7 | 1.7 | 1.7 | 1.9 | 1.5 | 1.7 | 1.7 | 2.1 | 0.6 | 0.7 | – |  |  |  |  |  |  |  |  |  |  |  |  |  |  |  |  |  |  |  |  |  |  |  |  |  |
| **12** | *Pristimantis* sp. 17_Ec_TungurahuaMachay_DHMECN14446 | 2.4 | 2.4 | 2.4 | 2.4 | 2.3 | 2.4 | 2.4 | 2.5 | 2.0 | 2.2 | 2.1 | – |  |  |  |  |  |  |  |  |  |  |  |  |  |  |  |  |  |  |  |  |  |  |  |  |
| **13** | *Pristimantis* sp. 17_Ec_TungurahuaMachay_DHMECN14450 | 2.4 | 2.4 | 2.4 | 2.4 | 2.3 | 2.4 | 2.4 | 2.5 | 2.1 | 2.2 | 2.1 | 0.0 | – |  |  |  |  |  |  |  |  |  |  |  |  |  |  |  |  |  |  |  |  |  |  |  |
| **14** | *Pristimantis* sp. 17_Ec_Nap_CordGuacamayos_QCAZ40808 | 2.2 | 2.2 | 2.2 | 2.4 | 2.1 | 2.2 | 2.3 | 2.6 | 1.9 | 2.1 | 1.9 | 0.4 | 0.4 | – |  |  |  |  |  |  |  |  |  |  |  |  |  |  |  |  |  |  |  |  |  |  |
| **15** | *Pristimantis* sp. 17_Ec_Nap_PactoSumaco_QCAZ41260 | 2.4 | 2.4 | 2.4 | 2.6 | 2.3 | 2.4 | 2.6 | 2.9 | 2.1 | 2.3 | 2.2 | 0.6 | 0.6 | 0.5 | – |  |  |  |  |  |  |  |  |  |  |  |  |  |  |  |  |  |  |  |  |  |
| **16** | *Pristimantis* sp. 17_Ec_Nap_PactoSumaco_QCAZ41305 | 2.4 | 2.4 | 2.4 | 2.6 | 2.3 | 2.4 | 2.6 | 2.9 | 2.1 | 2.3 | 2.2 | 0.6 | 0.6 | 0.5 | 0.2 | – |  |  |  |  |  |  |  |  |  |  |  |  |  |  |  |  |  |  |  |  |
| **17** | *P. festae*_Ec_Imb_LagunaPuruhanta_QCAZ11677 | 2.1 | 2.1 | 2.1 | 2.3 | 2.2 | 2.1 | 2.2 | 2.5 | 1.8 | 1.9 | 1.5 | 2.2 | 2.2 | 2.0 | 2.2 | 2.2 | – |  |  |  |  |  |  |  |  |  |  |  |  |  |  |  |  |  |  |  |
| **18** | *P. festae* _Ec_Imb_HdaZuleta_QCAZ52628 | 2.1 | 2.1 | 2.1 | 2.3 | 2.2 | 2.1 | 2.2 | 2.5 | 1.8 | 1.9 | 1.8 | 2.4 | 2.4 | 2.2 | 2.2 | 2.2 | 0.9 | – |  |  |  |  |  |  |  |  |  |  |  |  |  |  |  |  |  |  |
| **19** | *P. festae*_Ec_Imb_HdaZuleta_QCAZ52629 | 2.1 | 2.1 | 2.1 | 2.3 | 2.2 | 2.1 | 2.2 | 2.3 | 1.8 | 1.8 | 1.7 | 2.4 | 2.4 | 2.2 | 2.2 | 2.2 | 0.9 | 0.2 | – |  |  |  |  |  |  |  |  |  |  |  |  |  |  |  |  |  |
| **20** | *P. festae*_Ec_Pic_CayambeCoca_QCAZ49755 | 1.5 | 1.5 | 1.5 | 1.8 | 1.7 | 1.5 | 1.6 | 1.8 | 1.7 | 1.8 | 1.7 | 2.3 | 2.3 | 2.1 | 2.4 | 2.4 | 1.1 | 1.1 | 1.1 | – |  |  |  |  |  |  |  |  |  |  |  |  |  |  |  |  |
| **21** | *P. festae*_Ec_Car_LomaLaEsperanza_DBR3839 | 2.1 | 2.1 | 2.1 | 2.5 | 2.3 | 2.1 | 2.1 | 2.3 | 2.3 | 2.3 | 2.0 | 2.6 | 2.6 | 2.5 | 2.8 | 2.8 | 1.0 | 1.3 | 1.1 | 1.0 | – |  |  |  |  |  |  |  |  |  |  |  |  |  |  |  |
| **22** | *P. festae*_Ec_Car_SanFrancisco_DBR354 | 2.2 | 2.2 | 2.2 | 2.6 | 2.4 | 2.2 | 2.2 | 2.4 | 2.4 | 2.4 | 2.0 | 2.7 | 2.7 | 2.6 | 2.9 | 2.9 | 1.0 | 1.4 | 1.2 | 1.0 | 0.0 | – |  |  |  |  |  |  |  |  |  |  |  |  |  |  |
| **23** | *P. festae*_Ec_Car_SanFrancisco_DHMECN13640 | 2.2 | 2.2 | 2.2 | 2.5 | 2.4 | 2.2 | 2.3 | 2.4 | 2.1 | 2.2 | 2.0 | 2.5 | 2.5 | 2.4 | 2.6 | 2.6 | 1.2 | 1.5 | 1.5 | 1.1 | 0.2 | 0.2 | – |  |  |  |  |  |  |  |  |  |  |  |  |  |
| **24** | *P. festae*_Ec_Car_SanFrancisco_DHMECN13641 | 2.1 | 2.1 | 2.1 | 2.3 | 2.2 | 2.1 | 2.1 | 2.2 | 1.9 | 2.1 | 1.8 | 2.3 | 2.3 | 2.2 | 2.4 | 2.4 | 1.1 | 1.3 | 1.3 | 1.0 | 0.0 | 0.0 | 0.1 | – |  |  |  |  |  |  |  |  |  |  |  |  |
| **25** | *P. festae*_Ec_Imb_LagunaMojanda_MZUTI1807 | 2.2 | 2.2 | 2.2 | 2.4 | 2.4 | 2.2 | 2.3 | 2.3 | 1.9 | 2.1 | 1.5 | 2.2 | 2.2 | 2.1 | 2.4 | 2.4 | 0.8 | 1.3 | 1.3 | 1.4 | 1.0 | 1.0 | 1.4 | 1.2 | – |  |  |  |  |  |  |  |  |  |  |  |
| **26** | *P. festae*_Ec_Imb_LagunaMojanda_QCAZ13667 | 2.8 | 2.8 | 2.8 | 2.8 | 2.9 | 2.8 | 2.8 | 2.7 | 2.4 | 2.5 | 2.1 | 2.6 | 2.6 | 2.7 | 2.9 | 2.9 | 1.3 | 1.8 | 1.8 | 1.9 | 1.5 | 1.5 | 1.8 | 1.7 | 0.3 | – |  |  |  |  |  |  |  |  |  |  |
| **27** | *P. festae*_Ec_Imb_LagunaMojanda_QCAZ13677 | 2.6 | 2.5 | 2.5 | 2.5 | 2.7 | 2.5 | 2.6 | 2.5 | 2.2 | 2.3 | 1.8 | 2.3 | 2.3 | 2.4 | 2.7 | 2.7 | 1.1 | 1.6 | 1.5 | 1.7 | 1.3 | 1.4 | 1.7 | 1.5 | 0.1 | 0.1 | – |  |  |  |  |  |  |  |  |  |
| **28** | *P. festae*_Ec_Imb_LagunaMojanda_QCAZ42109 | 2.4 | 2.4 | 2.4 | 2.5 | 2.5 | 2.4 | 2.3 | 2.5 | 2.0 | 2.2 | 1.7 | 2.0 | 2.1 | 2.3 | 2.5 | 2.5 | 1.0 | 1.5 | 1.5 | 1.5 | 1.1 | 1.2 | 1.5 | 1.3 | 0.1 | 0.4 | 0.2 | – |  |  |  |  |  |  |  |  |
| **29** | *P. festae*_Ec_Imb_LagunaMojanda_QCAZ42116 | 2.4 | 2.4 | 2.4 | 2.5 | 2.5 | 2.4 | 2.3 | 2.5 | 2.0 | 2.2 | 1.7 | 2.0 | 2.1 | 2.3 | 2.5 | 2.5 | 1.0 | 1.5 | 1.5 | 1.5 | 1.1 | 1.2 | 1.5 | 1.3 | 0.1 | 0.4 | 0.2 | 0.0 | – |  |  |  |  |  |  |  |
| **30** | *P. festae*_Ec_Imb_13_8_km_W_Tabacundo_EF493515_KU218234) | 2.3 | 2.3 | 2.3 | 2.4 | 2.4 | 2.3 | 2.4 | 2.3 | 2.0 | 2.1 | 1.5 | 2.2 | 2.2 | 2.2 | 2.4 | 2.4 | 0.8 | 1.3 | 1.2 | 1.5 | 1.0 | 1.0 | 1.4 | 1.2 | 0.0 | 0.3 | 0.1 | 0.2 | 0.2 | – |  |  |  |  |  |  |
| **31** | *P. festae*_Ec_Car_ViaTulcanMaldonado_QCAZ43164 | 2.4 | 2.4 | 2.4 | 2.5 | 2.5 | 2.4 | 2.3 | 2.5 | 2.0 | 2.2 | 1.7 | 2.3 | 2.3 | 2.3 | 2.5 | 2.5 | 1.0 | 1.5 | 1.5 | 1.5 | 1.1 | 1.2 | 1.5 | 1.3 | 0.1 | 0.4 | 0.2 | 0.2 | 0.2 | 0.2 | – |  |  |  |  |  |
| **32** | *P. festae*_Ec_Nap_ParamoGuamani_QCAZ17950 | 2.6 | 2.5 | 2.5 | 2.5 | 2.7 | 2.5 | 2.6 | 2.5 | 2.2 | 2.3 | 1.8 | 2.3 | 2.3 | 2.4 | 2.7 | 2.7 | 1.3 | 1.8 | 1.7 | 1.7 | 1.5 | 1.5 | 1.6 | 1.5 | 0.6 | 0.6 | 0.6 | 0.7 | 0.7 | 0.6 | 0.7 | – |  |  |  |  |
| **33** | *P. festae*_Ec_Nap_ParamoGuamani_QCAZ49680 | 2.2 | 2.2 | 2.2 | 2.4 | 2.3 | 2.2 | 2.3 | 2.5 | 1.9 | 2.1 | 1.5 | 2.3 | 2.3 | 2.1 | 2.3 | 2.3 | 1.0 | 1.5 | 1.5 | 1.4 | 1.1 | 1.2 | 1.4 | 1.2 | 0.5 | 0.9 | 0.7 | 0.7 | 0.7 | 0.5 | 0.7 | 0.5 | – |  |  |  |
| **34** | *P. festae*_Ec_Nap_ParamoGuamani_QCAZ16405 | 2.3 | 2.3 | 2.3 | 2.5 | 2.4 | 2.3 | 2.3 | 2.5 | 1.9 | 2.1 | 1.6 | 2.3 | 2.3 | 2.2 | 2.4 | 2.4 | 1.1 | 1.6 | 1.6 | 1.4 | 1.1 | 1.0 | 1.4 | 1.2 | 0.5 | 0.9 | 0.7 | 0.6 | 0.6 | 0.5 | 0.6 | 0.5 | 0.2 | – |  |  |
| **35** | *P. festae*_Ec_Nap_ParamoGuamani_QCAZ16404 | 2.1 | 2.1 | 2.1 | 2.4 | 2.2 | 2.1 | 2.2 | 2.3 | 1.8 | 1.9 | 1.4 | 2.2 | 2.2 | 2.0 | 2.2 | 2.2 | 0.9 | 1.4 | 1.4 | 1.3 | 1.0 | 1.0 | 1.2 | 1.1 | 0.4 | 0.8 | 0.6 | 0.5 | 0.5 | 0.4 | 0.5 | 0.4 | 0.1 | 0.1 | – |  |
| **36** | *P. festae*_Ec_Pic_Cayambe_MZUTI4813 | 1.9 | 1.9 | 1.9 | 2.2 | 2.0 | 1.9 | 1.9 | 2.2 | 1.7 | 1.7 | 1.3 | 2.3 | 2.3 | 2.2 | 2.4 | 2.4 | 1.0 | 1.3 | 1.1 | 1.1 | 1.0 | 1.0 | 1.2 | 1.0 | 0.5 | 1.0 | 0.8 | 0.6 | 0.6 | 0.5 | 0.6 | 0.8 | 0.5 | 0.5 | 0.4 | – |

**Table S8.** Uncorrected genetic distance (16S), subclade 6 of *Pristimantis myersi* group.

|  | **Terminals** | **1** | **2** | **3** | **4** | **5** | **6** | **7** |
| --- | --- | --- | --- | --- | --- | --- | --- | --- |
| **1** | *Pristimantis* sp. 18_Ec_Ore_Dayuma_MH516183_QCAZ25589 | – |  |  |  |  |  |  |
| **2** | *Pristimantis* sp. 18_Ec_Car_Moran_DHMECN13332 | 0.2 | – |  |  |  |  |  |
| **3** | *Pristimantis* sp. 18_Ec_Car_LaBretana_DHMECN13328 | 0.2 | 0.0 | – |  |  |  |  |
| **4** | *Pristimantis* sp. 18_Ec_Car_51_3_km_W_Tulcan_EF493684_KU218227 | 0.2 | 0.3 | 0.3 | – |  |  |  |
| **5** | *Pristimantis* sp. 18_floridus_Ec_Car_Moran_DHMECN13336 | 6.0 | 3.6 | 3.7 | 3.9 | – |  |  |
| **6** | *Pristimantis* sp. 18_Ec_Car_LaCentella_TH639 | 6.6 | 6.8 | 6.8 | 6.6 | 7.1 | – |  |
| **7** | *Pristimantis* sp. 18_Ec_Car_LaCentella_TH668 | 4.4 | 2.7 | 3.2 | 3.2 | 5.8 | 5.2 | – |

**Table S9.** Uncorrected genetic distance (16S), subclade 7 of *Pristimantis myersi* group.

|  | **Terminals** | **1** | **2** | **3** | **4** | **5** | **6** | **7** | **8** | **9** | **10** | **11** | **12** | **13** | **14** | **15** | **16** | **17** | **18** | **19** | **20** | **21** | **22** | **23** | **24** | **25** | **26** | **27** | **28** | **29** | **30** | **31** | **32** | **33** | **34** | **35** | **36** | **37** | **38** |  |
| --- | --- | --- | --- | --- | --- | --- | --- | --- | --- | --- | --- | --- | --- | --- | --- | --- | --- | --- | --- | --- | --- | --- | --- | --- | --- | --- | --- | --- | --- | --- | --- | --- | --- | --- | --- | --- | --- | --- | --- | --- |
| **1** | *Pristimantis hectus*_Ec_Car_Dracula_DHMECN14993 | – |  |  |  |  |  |  |  |  |  |  |  |  |  |  |  |  |  |  |  |  |  |  |  |  |  |  |  |  |  |  |  |  |  |  |  |  |  |  |
| **2** | *P. hectus*_Ec_Car_Dracula_DHMECN14994 | 0.2 | – |  |  |  |  |  |  |  |  |  |  |  |  |  |  |  |  |  |  |  |  |  |  |  |  |  |  |  |  |  |  |  |  |  |  |  |  |  |
| **3** | *P. hectus* _Ec_Car_Dracula_DHMECN15165 | 0.2 | 0 | – |  |  |  |  |  |  |  |  |  |  |  |  |  |  |  |  |  |  |  |  |  |  |  |  |  |  |  |  |  |  |  |  |  |  |  |  |
| **4** | *P. hectus* _Ec_Car_Dracula_DHMECN14991 | 0.2 | 0.0 | 0.0 | – |  |  |  |  |  |  |  |  |  |  |  |  |  |  |  |  |  |  |  |  |  |  |  |  |  |  |  |  |  |  |  |  |  |  |  |
| **5** | *P. hectus* _Ec_Car_Dracula_DHMECN15172 | 0.2 | 0.0 | 0.0 | 0.0 | – |  |  |  |  |  |  |  |  |  |  |  |  |  |  |  |  |  |  |  |  |  |  |  |  |  |  |  |  |  |  |  |  |  |  |
| **6** | *P. hectus* _Ec_Car_Dracula_DHMECN15167 | 0.3 | 0.2 | 0.3 | 0.2 | 0.0 | – |  |  |  |  |  |  |  |  |  |  |  |  |  |  |  |  |  |  |  |  |  |  |  |  |  |  |  |  |  |  |  |  |  |
| **7** | *P. hectus* _Ec_Car_Dracula_DHMECN15169 | 0.2 | 0.0 | 0.0 | 0.0 | 0.0 | 0.0 | – |  |  |  |  |  |  |  |  |  |  |  |  |  |  |  |  |  |  |  |  |  |  |  |  |  |  |  |  |  |  |  |  |
| **8** | *P. hectus* _Ec_Car_RioChinambi_DHMECN14888 | 0.5 | 0.3 | 0.5 | 0.3 | 0.3 | 0.7 | 0.4 | – |  |  |  |  |  |  |  |  |  |  |  |  |  |  |  |  |  |  |  |  |  |  |  |  |  |  |  |  |  |  |  |
| **9** | *P. hectus* _Ec_Car_RioChinambi_DHMECN14847 | 0.7 | 0.5 | 0.5 | 0.5 | 0.5 | 0.5 | 0.5 | 0.2 | – |  |  |  |  |  |  |  |  |  |  |  |  |  |  |  |  |  |  |  |  |  |  |  |  |  |  |  |  |  |  |
| **10** | *P. hectus* _Ec_Car_RioChinambi_DHMECN14861 | 0.7 | 0.5 | 0.7 | 0.5 | 0.3 | 0.3 | 0.3 | 0.3 | 0.2 | – |  |  |  |  |  |  |  |  |  |  |  |  |  |  |  |  |  |  |  |  |  |  |  |  |  |  |  |  |  |
| **11** | *P. hectus* _Ec_Car_RioChinambi_DHMECN14887 | 0.7 | 0.5 | 0.7 | 0.5 | 0.3 | 0.3 | 0.3 | 0.3 | 0.2 | 0.0 | – |  |  |  |  |  |  |  |  |  |  |  |  |  |  |  |  |  |  |  |  |  |  |  |  |  |  |  |  |
| **12** | *P. onorei*_Ec_Pic_LasGralarias_MZUTI2023 | 4.5 | 4.3 | 4.5 | 4.3 | 4.1 | 3.1 | 3.7 | 4.3 | 3.9 | 3.0 | 3.0 | – |  |  |  |  |  |  |  |  |  |  |  |  |  |  |  |  |  |  |  |  |  |  |  |  |  |  |  |
| **13** | *P. onorei* _Ec_Pic_LasGralarias_MZUTI2030 | 5.4 | 5.1 | 5.4 | 5.1 | 4.7 | 3.0 | 4.0 | 5.0 | 4.3 | 2.8 | 2.8 | 0.2 | – |  |  |  |  |  |  |  |  |  |  |  |  |  |  |  |  |  |  |  |  |  |  |  |  |  |  |
| **14** | *P. onorei* _Ec_Pic_LasGralarias_MZUTI1465 | 4.7 | 4.6 | 4.7 | 4.7 | 4.4 | 3.7 | 4.3 | 4.7 | 4.5 | 3.4 | 3.4 | 0.3 | 0.4 | – |  |  |  |  |  |  |  |  |  |  |  |  |  |  |  |  |  |  |  |  |  |  |  |  |  |
| **15** | *P. onorei* _Ec_Pic_LasGralarias_MZUTI1464 | 4.7 | 4.6 | 4.7 | 4.7 | 4.4 | 3.7 | 4.3 | 4.7 | 4.5 | 3.4 | 3.4 | 0.3 | 0.4 | 0.0 | – |  |  |  |  |  |  |  |  |  |  |  |  |  |  |  |  |  |  |  |  |  |  |  |  |
| **16** | *P. onorei* _Ec_Pic_LasGralarias_MZUTI1467 | 4.4 | 4.2 | 4.4 | 4.4 | 4.1 | 3.5 | 4.0 | 4.4 | 4.1 | 3.1 | 3.2 | 0.6 | 0.7 | 0.5 | 0.5 | – |  |  |  |  |  |  |  |  |  |  |  |  |  |  |  |  |  |  |  |  |  |  |  |
| **17** | *P. onorei* _Ec_Pic_LasGralarias_MZUTI2037 | 5.7 | 5.5 | 5.7 | 5.5 | 5.3 | 4.1 | 5.0 | 5.5 | 5.3 | 4.0 | 4.0 | 0.9 | 0.4 | 1.1 | 1.1 | 0.8 | – |  |  |  |  |  |  |  |  |  |  |  |  |  |  |  |  |  |  |  |  |  |  |
| **18** | *P. onorei* _Ec_Pic_LasGralarias_MZUTI2038 | 5.8 | 5.7 | 5.8 | 5.1 | 5.5 | 5.1 | 6.2 | 5.1 | 6.4 | 4.2 | 4.3 | 0.8 | 0.2 | 2.1 | 2.1 | 2.1 | 0.1 | – |  |  |  |  |  |  |  |  |  |  |  |  |  |  |  |  |  |  |  |  |  |
| **19** | *P. onorei* _Ec_Pic_LasGralarias_MZUTI2024 | 4.2 | 3.9 | 4.2 | 3.9 | 3.7 | 2.6 | 3.2 | 3.9 | 3.4 | 2.4 | 2.5 | 0.4 | 0.2 | 0.5 | 0.5 | 0.4 | 0.2 | 0.0 | – |  |  |  |  |  |  |  |  |  |  |  |  |  |  |  |  |  |  |  |  |
| **20** | *P. onorei* _Ec_Pic_Bellavista_DHMECN6812 | 4.4 | 4.2 | 4.4 | 4.4 | 4.1 | 3.5 | 4.0 | 4.4 | 4.1 | 3.1 | 3.2 | 0.6 | 0.4 | 0.2 | 0.2 | 0.2 | 0.8 | 1.9 | 0.2 | – |  |  |  |  |  |  |  |  |  |  |  |  |  |  |  |  |  |  |  |
| **21** | *P. onorei* _Ec_Pic_Bellavista_DHMECN6821 | 4.4 | 4.2 | 4.4 | 4.4 | 4.1 | 3.5 | 4.0 | 4.4 | 4.1 | 3.1 | 3.2 | 0.6 | 0.4 | 0.2 | 0.2 | 0.2 | 0.8 | 1.9 | 0.2 | 0.0 | – |  |  |  |  |  |  |  |  |  |  |  |  |  |  |  |  |  |  |
| **22** | *P. onorei* _Ec_Pic_Bellavista_DHMECN6815 | 4.4 | 4.2 | 4.4 | 4.4 | 4.1 | 3.5 | 4.0 | 4.4 | 4.1 | 3.1 | 3.2 | 0.6 | 0.4 | 0.2 | 0.2 | 0.2 | 0.8 | 1.9 | 0.2 | 0.0 | 0.0 | – |  |  |  |  |  |  |  |  |  |  |  |  |  |  |  |  |  |
| **23** | *P. onorei* _Ec_Pic_Bellavista_DHMECN6813 | 4.4 | 4.2 | 4.4 | 4.4 | 4.1 | 3.5 | 4.0 | 4.4 | 4.1 | 3.1 | 3.2 | 0.6 | 0.4 | 0.2 | 0.2 | 0.2 | 0.8 | 1.9 | 0.2 | 0.0 | 0.0 | 0.0 | – |  |  |  |  |  |  |  |  |  |  |  |  |  |  |  |  |
| **24** | *P. onorei* _Ec_Pic_LasGralarias_MZUTI2031 | 4.5 | 4.3 | 4.5 | 4.3 | 4.1 | 3.9 | 3.8 | 4.3 | 4.1 | 3.8 | 3.4 | 1.7 | 2.4 | 1.5 | 1.5 | 1.5 | 1.7 | 1.7 | 1.8 | 1.3 | 1.3 | 1.3 | 1.3 | – |  |  |  |  |  |  |  |  |  |  |  |  |  |  |  |
| **25** | *P. onorei* _Ec_Pic_LasGralarias_MZUTI2032 | 5.2 | 4.8 | 5.2 | 4.8 | 4.4 | 3.3 | 3.7 | 4.7 | 4.0 | 3.1 | 2.8 | 0.9 | 0.7 | 1.1 | 1.1 | 0.9 | 0.7 | 0.5 | 0.5 | 0.7 | 0.7 | 0.7 | 0.7 | 1.6 | – |  |  |  |  |  |  |  |  |  |  |  |  |  |  |
| **26** | *P. onorei* _Ec_Pic_LasGralarias_MZUTI2035 | 4.2 | 3.9 | 4.1 | 3.9 | 3.6 | 2.6 | 3.1 | 3.8 | 3.3 | 2.5 | 2.5 | 0.4 | 0.2 | 0.4 | 0.4 | 0.2 | 0.2 | 0.0 | 0.0 | 0.0 | 0.0 | 0.0 | 0.0 | 0.0 | 0.0 | – |  |  |  |  |  |  |  |  |  |  |  |  |  |
| **27** | *P. onorei* _Ec_Imb_Toisan_MZUTI4241 | 4.3 | 4.2 | 4.3 | 4.2 | 4.0 | 3.2 | 3.7 | 4.4 | 4.2 | 3.2 | 3.2 | 1.2 | 1.5 | 1.2 | 1.2 | 0.9 | 1.8 | 1.8 | 1.1 | 0.9 | 0.9 | 0.9 | 0.9 | 2.2 | 1.8 | 0.9 | – |  |  |  |  |  |  |  |  |  |  |  |  |
| **28** | *P. onorei* _Ec_Imb_Toisan_MZUTI4244 | 4.4 | 4.2 | 4.4 | 4.2 | 4.1 | 3.4 | 4.0 | 4.4 | 4.3 | 3.2 | 3.2 | 1.2 | 1.5 | 1.2 | 1.2 | 1.0 | 1.8 | 2.9 | 1.1 | 1.0 | 1.0 | 1.0 | 1.0 | 2.2 | 1.8 | 0.9 | 0.0 | – |  |  |  |  |  |  |  |  |  |  |  |
| **29** | *P. onorei* _Ec_Imb_Toisan_MZUTI4243 | 4.4 | 4.2 | 4.4 | 4.2 | 4.1 | 3.4 | 4.0 | 4.4 | 4.3 | 3.2 | 3.2 | 1.2 | 1.5 | 1.2 | 1.2 | 1.0 | 1.8 | 2.9 | 1.1 | 1.0 | 1.0 | 1.0 | 1.0 | 2.2 | 1.8 | 0.9 | 0.0 | 0.0 | – |  |  |  |  |  |  |  |  |  |  |
| **30** | *P. onorei* _Ec_Cot_Otonga_MZUTI2063 | 4.2 | 4.0 | 4.2 | 4.0 | 3.8 | 3.2 | 3.5 | 3.8 | 3.6 | 2.9 | 2.9 | 1.1 | 1.4 | 1.2 | 1.2 | 0.9 | 1.6 | 1.4 | 0.9 | 0.9 | 0.9 | 0.9 | 0.9 | 0.9 | 1.2 | 0.9 | 0.9 | 0.9 | 0.9 | – |  |  |  |  |  |  |  |  |  |
| **31** | *P. onorei* _Ec_Cot_Otonga_MZUTI2094 | 4.4 | 4.2 | 4.4 | 4.2 | 4.0 | 3.3 | 3.7 | 4.0 | 3.8 | 3.0 | 3.0 | 1.4 | 1.9 | 1.4 | 1.4 | 1.2 | 1.9 | 1.7 | 1.3 | 1.2 | 1.2 | 1.2 | 1.2 | 1.2 | 1.7 | 1.3 | 0.9 | 0.9 | 0.9 | 0.3 | – |  |  |  |  |  |  |  |  |
| **32** | *P. onorei* _Ec_Cot_Otonga_MZUTI2095 | 4.4 | 4.2 | 4.4 | 4.2 | 4.0 | 3.3 | 3.7 | 4.0 | 3.8 | 3.0 | 3.0 | 1.4 | 1.9 | 1.4 | 1.4 | 1.1 | 1.9 | 1.7 | 1.3 | 1.1 | 1.1 | 1.1 | 1.1 | 1.2 | 1.7 | 1.3 | 0.9 | 0.9 | 0.9 | 0.3 | 0.0 | – |  |  |  |  |  |  |  |
| **33** | *P. onorei* _Ec_Cot_Otonga_QCAZ12288 | 4.2 | 4.1 | 4.2 | 4.2 | 3.9 | 3.5 | 3.8 | 4.0 | 3.8 | 3.0 | 3.0 | 1.2 | 1.6 | 1.1 | 1.1 | 0.8 | 1.7 | 2.8 | 1.1 | 0.8 | 0.8 | 0.8 | 0.8 | 1.7 | 1.9 | 1.1 | 0.7 | 0.8 | 0.8 | 0.1 | 0.1 | 0.1 | – |  |  |  |  |  |  |
| **34** | *P. onorei* _Ec_Cot_Otonga_MZUTI2070 | 4.4 | 4.2 | 4.4 | 4.2 | 4.0 | 3.3 | 3.7 | 4.0 | 3.8 | 3.0 | 3.0 | 1.3 | 1.6 | 1.3 | 1.3 | 1.0 | 1.7 | 1.6 | 1.1 | 1.0 | 1.0 | 1.0 | 1.0 | 1.0 | 1.4 | 1.1 | 0.7 | 0.7 | 0.7 | 0.1 | 0.1 | 0.1 | 0.0 | – |  |  |  |  |  |
| **35** | *P. onorei* _Ec_Cot_Otonga_MZUTI2088 | 4.4 | 4.2 | 4.4 | 4.2 | 4.0 | 3.3 | 3.7 | 4.0 | 3.8 | 3.0 | 3.0 | 1.3 | 1.6 | 1.3 | 1.3 | 1.0 | 1.7 | 1.6 | 1.1 | 1.0 | 1.0 | 1.0 | 1.0 | 1.0 | 1.4 | 1.1 | 0.7 | 0.7 | 0.7 | 0.1 | 0.1 | 0.1 | 0.0 | 0.0 | – |  |  |  |  |
| **36** | *P. onorei* _Ec_Cot_Otonga_MZUTI2093 | 4.4 | 4.2 | 4.4 | 4.2 | 4.0 | 3.3 | 3.7 | 4.0 | 3.8 | 3.0 | 3.0 | 1.3 | 1.6 | 1.3 | 1.3 | 1.0 | 1.7 | 1.6 | 1.1 | 1.0 | 1.0 | 1.0 | 1.0 | 1.0 | 1.4 | 1.1 | 0.7 | 0.7 | 0.7 | 0.1 | 0.1 | 0.1 | 0.0 | 0.0 | 0.0 | – |  |  |  |
| **37** | *P. onorei* _Ec_Cot_Otonga_MZUTI2075 | 4.4 | 4.2 | 4.4 | 4.2 | 4.0 | 3.3 | 3.7 | 4.0 | 3.8 | 3.0 | 3.0 | 1.3 | 1.6 | 1.3 | 1.3 | 1.0 | 1.7 | 1.6 | 1.1 | 1.0 | 1.0 | 1.0 | 1.0 | 1.0 | 1.4 | 1.1 | 0.7 | 0.7 | 0.7 | 0.1 | 0.1 | 0.1 | 0.0 | 0.0 | 0.0 | 0.0 | – |  |  |
| **38** | *P. onorei* _Ec_Cot_Otonga_MZUTI2066 | 4.4 | 4.2 | 4.4 | 4.2 | 4.0 | 3.3 | 3.7 | 4.0 | 3.8 | 3.0 | 3.0 | 1.3 | 1.6 | 1.3 | 1.3 | 1.0 | 1.7 | 1.6 | 1.1 | 1.0 | 1.0 | 1.0 | 1.0 | 1.0 | 1.4 | 1.1 | 0.7 | 0.7 | 0.7 | 0.1 | 0.1 | 0.1 | 0.0 | 0.0 | 0.0 | 0.0 | 0.0 | – |  |
| **39** | *P. onorei* _Ec_Cot_Otonga_MZUTI2092 | 4.4 | 4.2 | 4.4 | 4.2 | 4.0 | 3.3 | 3.7 | 4.0 | 3.8 | 3.0 | 3.0 | 1.3 | 1.6 | 1.3 | 1.3 | 1.0 | 1.7 | 1.6 | 1.1 | 1.0 | 1.0 | 1.0 | 1.0 | 1.0 | 1.4 | 1.1 | 0.7 | 0.7 | 0.7 | 0.1 | 0.1 | 0.1 | 0.0 | 0.0 | 0.0 | 0.0 | 0.0 | 0.0 | – |
